# Shortcut Access to Peptidosteroid Conjugates: Building Blocks for Solid-Phase Bile Acid Scaffold Decoration by Convergent Ligation

**DOI:** 10.3390/molecules161210168

**Published:** 2011-12-07

**Authors:** Dieter Verzele, Sara Figaroli, Annemieke Madder

**Affiliations:** Laboratory for Organic and Biomimetic Chemistry, Department of Organic Chemistry, Faculty of Sciences, Ghent University, B-9000 Ghent, Belgium

**Keywords:** bile acids, solid-phase synthesis, scaffold decoration, peptidosteroids, convergent ligation

## Abstract

We present three versatile solid-supported scaffold building blocks based on the (deoxy)cholic acid framework and decorated with handles for further derivatization by modern ligation techniques such as click chemistry, Staudinger ligation or native chemical ligation. Straightforward procedures are presented for the synthesis and analysis of the steroid constructs. These building blocks offer a new, facile and shorter access route to bile acid-peptide conjugates on solid-phase with emphasis on heterodipodal conjugates with defined spatial arrangements. As such, we provide versatile new synthons to the toolbox for bile acid decoration.

## 1. Introduction

**Figure 1:**
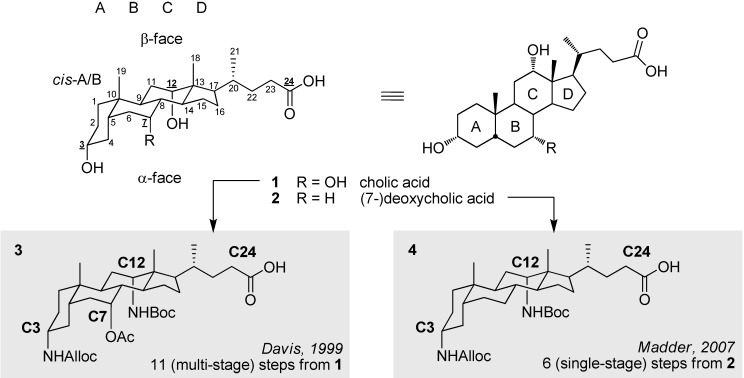
Selection of natural bile acids and related synthetic amino derivatives relevant for current work.

Among the variety of molecular scaffolds employed in supramolecular chemistry [1,2,3], steroids in general, and bile acids in particular, have received a great deal of attention over the last decades [4,5,6,7,8]. The interest in these latter natural products is explained by their unique combination of rigidity and chirality, high availability, biocompatibility, and the various functionalization patterns that can be modified in a tunable manner. Distributed around a tetracyclic framework as shown in Figure 1, the well-spaced array of selectively addressable moieties makes molecules such as 3α,7α,12α-trihydroxy-5β-cholan-24-oic acid (cholic acid, **1**) and 3α,12α-dihydroxy-5β-cholan-24-oic acid (7-deoxycholic acid, **2**) versatile synthons to develop pre-organized conjugates for applications based on cooperativity. The *cis*-A/B ring junction imparts a curved cavity profile, and assists in differentiation between the hydroxyl groups. The A, B, C and D (cyclopentano)perhydrophenanthrene ring structures define two planes, generally referred to as the α- and β-face, and explaining their so-called facial amphiphilicity (a convex/hydrophobic β-face and a concave/hydrophilic α-face, combined with a negatively-charged side chain). Whereas the cholanic skeleton of bile acids is naturally endowed with a *cis* A/B-ring fusion (*i.e.*, 5β-configuration), *trans* isomers resulting in allocholanic acids (*i.e.*, 5α-configuration) can be obtained synthetically. The naturally present spacer and carboxylic acid group at the C24 position allow for convenient immobilization to a solid-phase resin and/or further derivatization with a variety of moieties. Hirschmann *et al*. stipulated a correspondence between the steroid backbone and a cyclic hexapeptide scaffold, yet avoiding the inconveniencies of applying naturally occurring peptides [9,10]. Additionally, conjugates with improved pharmacological profiles in terms of bioavailability and biostability demonstrate the further potential of modified bile acids as so-called Trojan Horse carriers in drug discovery [6,11,12,13,14,15,16].

The group of Still *et al*. pioneered the use of bile acids as molecular scaffolds for the generation of peptide libraries on solid-support [17,18,19]. Based on both naturally-occurring 3α,7α-dihydroxy-5β-cholan-24-oic acid (chenodeoxycholic acid) and the synthetically-prepared N-allo derivative (A,B-*trans*), two peptidosteroid libraries (each containing 10,000 members) were prepared via the combinatorial split-pool methodology. This approach was further elaborated by Wess *et al*. [20], Nestler [21], HØeg-Jensen [22] and Savage *et al*. [23]. 

The expanding number of applications employing bile acid templates has stimulated the development of new derivatives with improved properties. Depending on the structural requirements of the desired conjugates and the envisaged synthetic strategy, various scaffolds have been prepared. Generally, the initially-applied naturally-occurring bile acids fail to allow efficient application in a wide range of contemporary, advanced investigations. The obvious esterification of the natural hydroxyl functions proved slow and hard to perform consecutively in a reliable way and is therefore not ideally-suited for automated solid-phase procedures. The intrinsic lability of the ester moiety prevents further elaboration of extended or complex peptide assemblies, since repetitive treatment of the peptide reagents results in premature cleavage and/or side reactions. Therefore, in recent years, emphasis has been put on the replacement of the hydroxyl by amino functionalities, with tripodal scaffold **3** and dipodal counterpart **4** as state-of-the art members reported by Davis *et al*. [24] and our group [25,26], respectively. The amino groups are readily convertible into stable linkages, most often amides, and allow for reliable elaboration on solid supports. A more complete overview of all endeavors towards multipodal amino-based scaffolds has been earlier overviewed by the former group [27].

**Figure 2:**
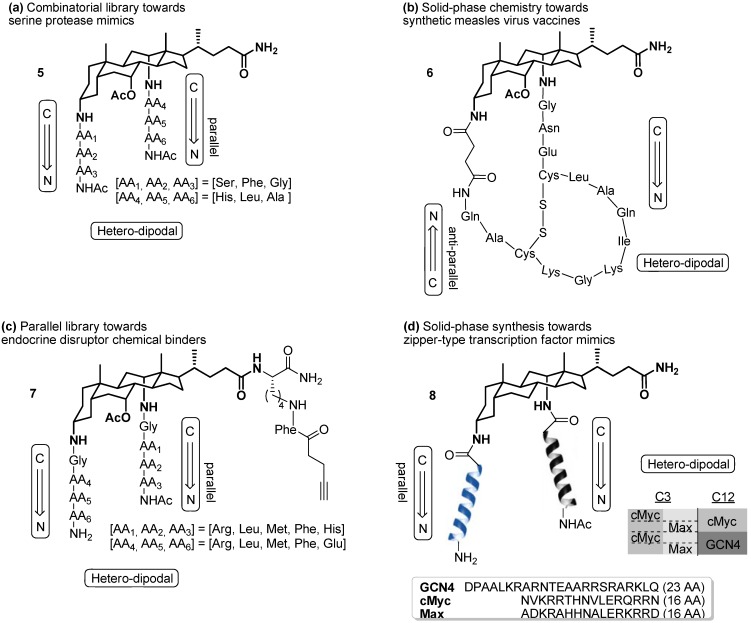
Bile acid-peptide conjugates contributed by our group.

In collaboration with us, scaffold **3** was used in a combinatorial search for serine protease-like activity via conjugate **5** by our own research group (Figure 2) [28,29]. This allowed for further generation of loop structures **6** (incorporating longer peptide sequences) as synthetic vaccines against the measles virus [30,31], complementing the few successful attempts towards preparation of both cyclic [15,20] and cyclodimeric peptidosteroid macrocycles [32,33,34,35] in literature. Most recently, the established methodologies allowed for the parallel solid-phase synthesis of a first generation of receptors **7** for endocrine disruptor chemicals (EDCs) [36], while the architectural features of the bile acid framework were further exploited in the development of zipper-type transcription factor miniatures by heterodimeric tweezer models **8**, based on building block **4** [37]. Whereas all constructs depicted in Figure 2 were synthesized by consecutive chain elongation through stepwise linear SPPS procedures, our current interest has shifted towards the possibilities of contemporary ligation schemes for the convergent assembly of our peptidosteroid targets on solid-phase.

We thus would like to expand the available repertoire with building blocks suitably decorated for subsequent chemoselective and convergent methodologies, aiming for shortcut access through modularity. To this purpose, we here disclose a series of carrier-supported bile acid scaffold building blocks accessible through facile, versatile methodologies.

## 2. Results and Discussion

### 2.1. Ultrashort Access towards a Template with (Limited) Ligation Properties

Considering the series of previously developed amino based scaffolds, orthogonal protection of the amino groups significantly increases the versatility of the scaffold, while stereocontrol at every position is required. However, further taking reactivity of the C24-functionality into account, synthesis of such compounds is not straightforward, especially considering that gram quantities are needed for most applications. The limited number of so-called ideal scaffolds such as compound **3** and dipodal counterpart **4** confirms their non-trivial preparation.

**Figure 3:**
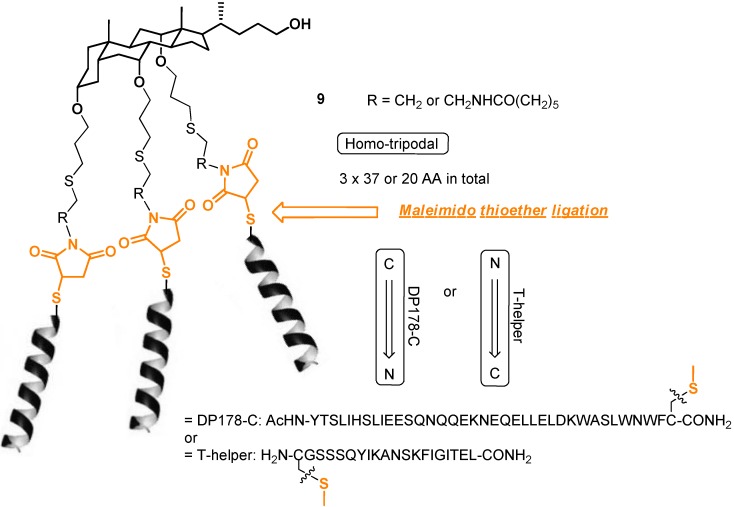
Macromolecular peptidosteroid ligation precedent by Wang *et al*. [38].

As for literature precedents on convergent ligation of OH-based bile acids, a cholic acid building block has previously been decorated through thioether ligation by Wang *et al*. in solution [38], using maleimide- or bromoacetyl moieties and yielding homotrimeric protein-like assembly **9** (Figure 3) [39].

Considering more recently developed convergent ligation strategies, we decided to explore the possibility of combining the here illustrated Wang alkylation methodology with a click chemistry or Staudinger ligation [40] approach in an attempt to develop a fast and easy access to a template suitable for double orthogonal convergent ligation. Indeed, in the course of our synthetic efforts towards amino based templates, various routes in literature were noticed to proceed via azide introduction at C3 and subsequent reduction to the desired amino functionality.

**Scheme 1:**
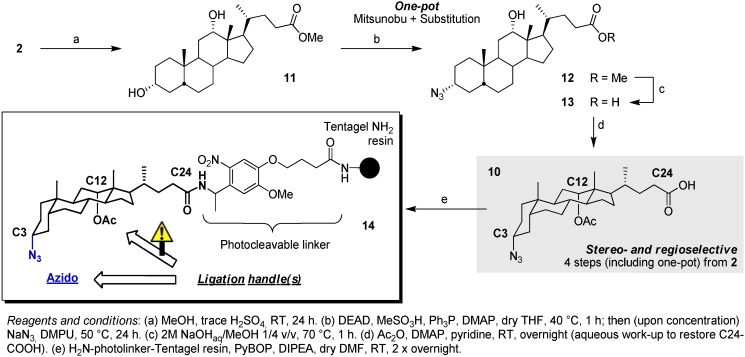
Synthesis of construct **14** as an initial attempt towards azido-based peptidosteroid ligations.

Starting from deoxycholic acid (**2**), we embarked on the ultrashort synthesis of analogue **10** that should allow for orthogonal double ligation (C3-N_3_ click + C12-OH alkylation). The complete synthetic strategy is outlined in Scheme 1. Simplicity of selective C3-azido introduction with defined stereochemistry at deoxycholic acid derivative **11** provides for straightforward introduction of the first click handle. In contrast to the literature where (partial) purification of the intermediate mesylate by flash chromatography is usually reported [41,42,43,44], we were able to shorten the introduction of the azide group by applying a genuine one-pot procedure. It was however necessary to heat the S_N_^2^ reaction up to 50 °C, whereas literature suggests a lower temperature (40 °C). Selective introduction of the azide at C3 can be explained by the fact that the equatorial C3α-group is less hindered than the axial 7α- and 12α-groups. Reactivity of the 12α-OH is further lowered by the neopentyl-like surrounding and the proximal C21/C18 methyl-groups. Subsequent C24-ester hydrolysis at intermediate **12** and C12-OH acetylation [45] at **13** can then be followed by immobilization on a suitable solid support. As illustrated in our previous work, immobilization through a photocleavable linker allows for straightforward analysis of intermediate adducts and final compounds after simple irradiation of resin samples. Resulting construct **14**, accessible in only 5 steps, should thus allow for double ligation through consecutive click and alkylation procedures.

Since its emergence 10 years ago [46], azide-alkyne triazole click chemistry rapidly became a reliable method for cholic acid derivatization, with applications as broad as the bile acid field itself [43,44,47,48,49,50]. Literature studies confirm that Cu catalyzed 1,3-dipolar cycloaddition at C3 can proceed smoothly and has previously also been illustrated for attachment of peptide chains [49].

However, as for the further modification of C12-OH, though examples exist, it is known that the natural hydroxyl groups are relatively unreactive and often show troublesome derivatization, especially the axial 7- and 12-OH. Correspondingly, further decoration of the C12-OH proved non-trivial in our hands and despite literature precedents, all our preliminary attempts to alkylate failed. Conversion of this hydroxyl group to the corresponding carbamates upon isocyanate treatment allows for more rapid derivatization, yet lacks versatility (and stability; an acid-labile Boc-like moiety is generated at the C12 position). Furthermore, though acylation of the C12-OH is possible, this inevitably causes the presence of base labile ester linkages. Therefore, though the current scaffold structure can be useful in some applications, we continued our studies towards a more universally applicable double convergent ligation template. In what follows we relied on the firm methodology of Davis and Madder amino based building blocks, established during the development of previously mentioned in-house constructs.

### 2.2. From EDC Receptors to a Simple Azide/Alkyne-Decorated Building Block for Click and Staudinger Ligation

**Scheme 2:**
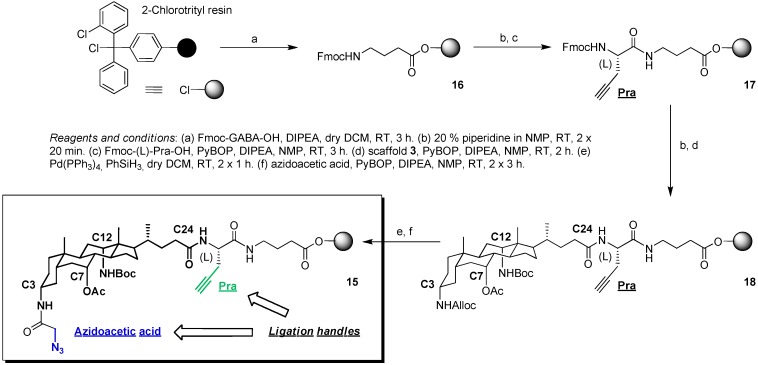
Synthesis of construct **15** as shortcut option towards peptidosteroid conjugates via click/Staudinger ligation.

Further attracted by the popularity of the contemporary Huisgen click and Staudinger ligation chemistries, more interesting opportunities in that direction were found during synthesis of the aforementioned EDC sensor conjugates **7**, more specifically through one of the intermediates featuring an azide functionality as orthogonal protection for the corresponding amine and an alkyne moiety for later screening purposes [36]. An example of such efforts towards template-assembled multivalent triazole conjugates by decorating a cyclic decameric peptide scaffold has been recently contributed by Avrutina *et al*. [51]. In contrast to the usual reduction of a C3-azido(acetic acid) handle through Staudinger reduction (before linear SPPS elongation) both here and in other contributions, exploitation of the ligation variant occurred to us as a more efficient route for scaffold decoration. While maintaining the obvious role of the terminal alkyne incorporated at the C24-linker position (Figure 2, structure **7**), a new bile acid building block amenable for double ligation on solid-support was envisaged. At the same time, as improved model for the estrogen receptor hormone binding domain (ERHBD), the enlarged binding cavity featuring an increased distance between the anchor points might enhance the performance of our receptor candidates for EDC accommodation in contrast to the original C3α-C12α organization. Though of potential benefit in the specific case of EDC receptors with potential induced fit properties, the concurrent loss of rigidity might not always be desirable. While in first instance, an alkyne moiety of the type included in structure **7** was considered, doubts arose about the possible interference of intramolecular cyclization between the alkyne and an azide moiety introduced at the cholic acid framework, due to the length of the external linker as can be observed in Figure 2. Such an event could be hard to trace, since mass spectrometric detection would fail to discriminate between the cyclized side-product and the starting material due to the atom efficiency of the click reaction, by definition. To avoid such complications it was decided to prepare a modified counterpart **15**, with a shorter propargylglycine unit as alkyne linker (Scheme 2).

In a first step, Fmoc-protected γ-aminobutyric acid (GABA) was immobilized on the solid support as spacer to yield **16**. Upon Fmoc removal, Fmoc protected L-propargylglycine-OH was coupled as external alkyne linker. Resulting spacer **17** was deprotected, yielding **18** upon coupling of building block **3**. Developed by Davis *et al*. and also used in the synthesis of both **5** and **6**, a unique feature is the straightforward design of not only homodimers, but also heterodimeric counterparts due to orthogonal N-protection, in contrast to the homomeric precedents by Wang and Avrutina. Considering that in the envisaged convergent strategy the C12-Boc protecting group on the scaffold will not be removed, 2-Chlorotrityl resin was suitable as solid support. Due to the high loading value (1.55 mmol/g) considerable amounts of product can be obtained, while its acid lability guarantees efficient detachment of the products. Upon Alloc deprotection, azidoacetic acid was smoothly coupled to provide the azide functionality and complete the synthesis of our second ligation template **15**. Optimized earlier [36], Alloc deprotection was achieved using phenyl silane (PhSiH_3_, 25 eq.) as allyl group scavenger, combined with Pd(0) tetrakistriphenylphosphine [Pd(PPh_3_)_4_, 0.1 eq.] as catalyst.

In view of the shorter length of the alkyne bearing chain and combined with the large resulting distance between azide *vs.* alkyne moieties, competitive intramolecular click reactions can be excluded and straightforward scaffold decoration can start from here. 

### 2.3. From Zipper-Type Protein Miniatures to Cys-Decorated Building Block for Double Orthogonal, Interthiol Assisted Native Chemical Ligation

While the above route furnishes a shortcut option towards peptidosteroids by exploiting the SPPS handles for ligation purposes, shortcuts can also be made on the level of the basic, undecorated scaffold building block. Previously discussed scaffold **15** was constructed starting from the C3-NHAlloc/C7-OAc/C12-NHBoc derivative **3** conceived by Davis *et al*. Although orthogonal protection adds greatly to the versatility of bile acid scaffolds, differentiation between the axial 7- and 12-positions is very difficult [27]. During synthesis of such highly differentiated templates, sequential derivatization is often performed in separate steps to maximize configurational control, correct differentiation and to minimize the need to separate diastereomeric mixtures of highly polar polyamine derivatives. Despite the application of various selective conversions, these synthetic routes require extensive steps and chromatographic separation at several stages. Therefore, large-scale preparation is tedious, time-consuming and as such not ideal for routine use [24]. As mentioned above, in-house application of this scaffold has resulted in the generation of combinatorial libraries and cyclic peptidosteroids on solid-support. Yet the dipodal application of this essentially tripodal scaffold is far from logical. While differentiation between three in lieu of two functionalities substantially complicates and lengthens the synthetic route, the 7-OAc has very limited application for further elaboration. Moreover, this essentially passive moiety proved reactive under certain conditions, leading to side-products accumulating on the solid-phase resin. Next to suitable geometric properties discussed earlier, the use of derivative **4**, lacking the fractious functionality at the C7-position, was the obvious alternative. Surprisingly, preparation of this scaffold had not been reported in literature. The lack of rapid, large-scale preparations for suitably-protected, dipodal scaffolds with desired stereochemistry in literature prompted the development of compound **4**, complementing published analogues.

Whereas the strategy described above used building block **3** synthesized by Davis *et al*. in 11 (multi-stage) steps from cholic acid (**1**), the simpler version **4** (Figure 1) of this essentially dipodal scaffold has been obtained in our group starting from deoxycholic acid (**2**) through an ultra short 6-step synthetic route [25].

As illustrated in the introduction this was exploited in the development of zipper type transcription factor miniatures (Figure 2 above, structure **8**) [37]. However, linear SPPS procedures for such protein-like macromolecular conjugates easily become long and cumbersome owing to aggregation phenomena of the growing peptide chain with the already present one immobilized in forced proximity. Therefore proceeding towards alternative synthesis routes through the widely-employed native chemical ligation methodology by Kent *et al*. [52] seems a viable alternative. Apart from the aforementioned maleimido/bromoacetyl thioether and triazole click ligations on bile acids, to the best of our knowledge, few to none further attempts in that direction have been published thus far, and suitable building blocks have neither been reported in turn.

**Scheme 3:**
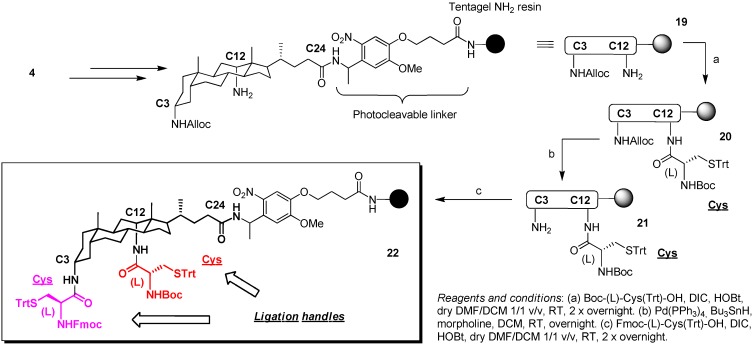
Synthesis of construct **22** as shortcut option towards peptidosteroid conjugates via double orthogonal, interthiol assisted native chemical ligation.

Starting from the aforementioned contribution, scaffold **4** was immobilized onto a Tentagel-photolinker yielding construct **19 **(Scheme 3). Subsequent coupling of NHBoc-Cys(STrt) through conditions optimized by Albericio *et al*. [53], instead of the usual PyBOP chemistry in DMF or NMP, allowed us to avoid stereomutation of this epimerization-prone residue to furnish intermediate **20**. Similar as above [36] and earlier findings in our lab [26], case-by-case optimization of Alloc deprotection was again necessary. As proven by ESI-MS, LC-MS and RPHPLC, repeated treatment of **20** with anilinium *p*-toluenesulfinate (20 eq.) + Pd(PPh_3_)_4_ (0.15 eq.) in NMP (400 μL) for 2 h at room temperature (Ar, shielded from light) failed to remove the C3-NHAlloc protecting group of the resin-supported scaffold. Single application of morpholine (180 eq.) + Bu_3_SnH (20 eq.) + Pd(PPh_3_)_4_ (0.2 eq.) in DCM (3 mL) at room temperature (Ar, shielded from light) also failed in deprotection. Eventual conversion was achieved by using a slight excess of Pd(PPh_3_)_4_ instead of catalytic amounts, presumably due to poisoning of the Pd catalyst by the sulfur atoms, and/or hindrance by the bulky trityl-group rigidly affixed in close proximity on the steroid core. Upon similar coupling of NHFmoc-Cys(STrt) on resulting **21**, scaffold **22** for double orthogonal NCL chemistry was obtained. Indeed, initial removal of the Boc group to start with the usual decoration of the 12 position also liberates the Trt at the C3-moiety, which cannot proceed in NCL because of the Fmoc-shielded amine. Hence, again access is granted towards heterodimeric ligation products. The enforced vicinity of this Cys side chain at the C3 position might further assist in decoration of the adjacent C12-moiety.

Both in terms of synthesis and design, the convergent approach provides at the same time alternative, efficient and modular access to enhanced conjugates, increase of the ~7–8 Å spacing of the co-directional attachment points, and alleviation of steric impediment by the close restriction between them on the rigid steroid core.

## 3. Experimental

### 3.1. General Information

DCM was distilled from CaH_2_ when used dry (for Alloc removal or coupling steps). Dry THF was distilled from sodium benzophenone ketyl. The H_2_N-photolinker-Tentagel resin [37], scaffold **3** [24], azidoacetic acid [54] and resin **19** [37] were synthesized according to literature prior to this work. All other reagents and solvents were obtained from commercial suppliers and used without further purification. Redistilled 99.5% pure DIPEA has been used throughout, whereas extra dry DMF was purchased on molecular sieves (water < 50 ppm) when used for coupling reactions. In all other cases, DMF as well as NMP were peptide grade quality, while all other solvents were HPLC grade quality. 2-Chlorotrityl resin was specified as 1.55 mmol/g, 74–149 µm. All reactions were performed under argon atmospheres. Analytical TLC was carried out on glass plates precoated with silica gel (60F254, 0.25 mm). Compounds were visualized by phosphomolybdic acid (PMA). Flash chromatography was performed on Kieselgel Merck Typ 9385 230–400 mesh, 60 Å. NMR spectra were recorded in CDCl_3_ (99.8 atom% D) at room temperature on a Bruker Avance 300 or 500 spectrometer at 300 or 500 MHz for ^1^H-NMR and 75 or 125 MHz for ^13^C-NMR spectra. Chemical shifts (*δ* units) are expressed in parts per million (ppm) relative to tetramethylsilane (TMS) and the internal solvent peak was used for calibration. When peak multiplicities are reported, the following abbreviations are used: s, singlet; d, doublet; m, multiplet; br, broad. Coupling constants (*J* values) are expressed in Hertz (Hz). The Attached Proton Test (APT) technique was used to assign ^13^C peaks (C, CH, CH_2_, CH_3_). All ^1^H-NMR spectra of (deoxy)cholic acid derivatives contain a region of high signal overlap between ~1.00 and ~2.00 ppm, which is generally referred to as a series of multiplets and therefore not listed in the spectral details. Fortunately, in the preparation of compound **10**, the important signals are shifted downfield from this region and included in the assignments below. Furthermore, ^13^C-APT spectra proved well-resolved and diagnostic. UV-Fmoc loading determination was performed with a Varian Cary 300 Bio UV-VIS spectrophotometer. ESI-MS spectra were recorded on a quadrupole ion trap LC mass spectrometer (Thermo Finnigan MAT LCQ), equipped with electrospray ionization. MeOH/H_2_O (4/1 ± 0.1% formic acid or 5 mM NH_4_OAc) was used as carrier solution. Reversed-Phase LC-MS analysis was performed on an Agilent 1100 Series HPLC instrument equipped with a Phenomenex Luna C18 (2) 100 Å column (250 × 4.6 mm, 5 μ, at 35 °C) using a flow = 1.0 mL/min and coupled to an Agilent ESI-single quadrupole MS detector type VL. By using a binary solvent system composed of 5 mM NH_4_OAc in H_2_O (A) and ACN (B) as mobile phases, linear gradient elution has been performed. The column was flushed for 2 min with 0% B, then a gradient from 0 to 100% B over 15 min was applied, followed by 5 min of flushing with 100% B, after which the gradient returns to 0% B in 0.5 min, concluding the cycle by flushing for 3 min. Reversed-Phase HPLC (RPHPLC) analysis was performed on an Agilent 1100 Series instrument equipped with a Phenomenex Luna C18 (2) 100 Å column (250 × 4.6 mm, 5 μ, at 35 °C) and using a flow = 1.0 mL/min. By using a binary solvent system composed of 0.1% TFA in H_2_O (A) and ACN (B) as mobile phases, linear gradient elution has been performed. The column was flushed for 3 min with 0 or 75% B, then a gradient from 0 or 75 to 100% B over 15 min was applied, followed by 5 min of flushing with 100% B, after which the gradient returns to the starting composition in 0.5 min, concluding the cycle by flushing for 3 min. Relevant spectra and chromatograms are further included as Supporting Information.

### 3.2. Synthesis of Scaffold ***14***

*Esterification of deoxycholic acid* (**2**)*→ Methyl 3α,12α-dihydroxy-5β-cholan-24-oate* (**11**): Deoxycholic acid (10 g, 25.685 mmol) was dissolved in MeOH (100 mL) to give a yellowish mixture, which became clear upon addition of a trace of H_2_SO_4_ (600 µL, 9.9 mmol, 0.4 eq.). The reaction mixture was stirred overnight at room temperature. The reaction was followed by TLC (isooctane/EtOAc 1/4, PMA) which showed complete consumption of starting material. The solvent was evaporated under reduced pressure and dried to obtain **11** (11.59 g, quantitative isolated yield) as a white solid. This product proved pure enough for further reaction. R_f_ (isooctane/EtOAc 1/4, PMA) 0.41. ^1^H-NMR (300 MHz, CDCl_3_) *δ* 4.06 (1H, br s, 12β-H), 3.75 (1H, m, 3β-H), 3.66 (3H, s, ester CH_3_), 2.43–2.31 (1H, br m), 2.30–2.16 (1H, br m), 0.96 (3H, d, *J* = 6.1, 21-CH_3_), 0.91 (3H, s, 19-CH_3_), 0.68 (3H, s, 18-CH_3_). ^13^C-NMR (APT, 125 MHz, CDCl_3_) *δ* 12.6 (18-CH_3_), 17.2 (21-CH_3_), 23.0 (19-CH_3_), 23.5 (CH_2_), 26.0 (CH_2_), 27.0 (CH_2_), 27.4 (CH_2_), 28.4 (CH_2_), 30.0 (CH_2_), 30.8 (CH_2_), 31.0 (CH_2_), 33.5 (CH), 34.0 (C), 35.0 (CH), 35.1 (CH_2_), 35.89 (CH), 35.92 (CH_2_), 42.0 (CH), 46.3 (C), 47.2 (CH), 48.1 (CH), 51.4 (ester CH_3_), 72.0 (12-CH), 73.3 (3-CH), 174.6 (COOR). ESI^+^-MS (250 °C, MeOH/H_2_O 4/1) calcd. for C_25_H_42_O_4_ 406.3 Da, found *m/z* (% rel. int.) 371.2 (100) [M − 2H_2_O + H]^+^, 388.8 (19) [M − H_2_O + H]^+^. The spectra are available in the Supporting Information: Figures S1–S3.

*One-pot Mitsunobu-substitution reaction at compound* **11** → *Methyl 3α-azido,12α-hydroxy-5β-cholan-24-oate* (**12**): Ester **11** (5.011 g, 12.324 mmol), PPh_3_ (9.679 g, 36.9 mmol, 3 eq.), DMAP (3.006 g, 24.61 mmol, 2 eq.) and MeSO_3_H (1.6 mL, 24.657 mmol, 2 eq.) were dissolved in dry THF (59.5 mL). The resulting white mixture was heated at 40 °C and DEAD (6.8 mL, 37.1 mmol, 3 eq.) was slowly added, turning the reaction orange. TLC (isooctane/EtOAc 1/4, PMA) showed complete conversion of starting material after 1 h of stirring. The solvent was evaporated under reduced pressure, the residue dried at high vacuum and the resulting crude directly used for S_N_^2^ reaction. Redissolved in DMPU (35 mL), NaN_3_ (6.009 g, 92.43 mmol, 7.5 eq.) was added and the reaction mixture heated at 50 °C for 24 h. After TLC (hexane/EtOAc 1/1, PMA) showed complete consumption of the intermediate mesylate, compound **12** was extracted into CHCl_3_ and washed with brine. The combined CHCl_3_ isolates were concentrated under reduced pressure. The residue was redissolved in hexane/EtOAc 2/1 and filtered through a patch of silica, followed by concentration under reduced pressure. After flash chromatography (hexane/EtOAc 9/1), pure **12** was obtained as white solid (2.685 g, 50.6% isolated yield). R_f _(hexane/EtOAc 9/1, PMA) 0.16. ^1^H-NMR (300 MHz, CDCl_3_) *δ* 3.98 (1H, br s, 12β-H), 3.67 (3H, s, ester CH_3_), 3.33 (1H, m, 3β-H), 2.43–2.31 (1H, br m), 2.30–2.18 (1H, br m), 0.97 (3H, d, *J* = 6.2, 21-CH_3_), 0.92 (3H, s, 19-CH_3_), 0.67 (3H, s, 18-CH_3_). ^13^C-NMR (APT, 75 MHz, CDCl_3_) *δ* 12.8 (18-CH_3_), 17.3 (21-CH_3_), 23.3 (19-CH_3_), 23.6 (CH_2_), 26.0 (CH_2_), 26.7 (CH_2_), 27.0 (CH_2_), 27.4 (CH_2_), 28.7 (CH_2_), 30.9 (CH_2_), 31.1 (CH_2_), 32.5 (CH_2_), 33.7 (CH), 34.2 (C), 35.1 (CH), 35.4 (CH_2_), 36.0 (CH), 42.4 (CH), 46.5 (C), 47.4 (CH), 48.2 (CH), 51.5 (ester CH_3_), 61.3 (3-CH), 73.1 (12-CH), 174.7 (COOR). ESI^+^-MS (250 °C, MeOH/H_2_O 4/1 + 0.1% formic acid) calcd. for C_25_H_41_N_3_O_3_ 431.3 Da, found *m/z* (% rel. int.) 386.1 (100) [M − N_2_ − OH]^+^, 454.0 (92) [M + Na]^+^. The spectra are available in the Supporting Information: Figures S4–S6.

*Basic hydrolysis of compound ***12** → *3α-Azido,12α-hydroxy-5β-cholan-24-oic acid* (**13**): Compound **12** (100.3 mg, 0.232 mmol) was dissolved in MeOH (8 mL) in a round-bottomed flask (25 mL, + reflux cooler), which is flushed with argon. An aqueous NaOH solution (2 mL, 2 M) was added and the resulting white suspension stirred at 70 °C, showing complete redissolution of the reaction mixture. After 1 h, complete conversion was shown by TLC verification (hexane/EtOAc 4/1, PMA) and the reaction was cooled to room temperature. The MeOH solvent was evaporated under reduced pressure, the white residue transferred to a separation funnel (100 mL) and this aqueous suspension acidified (pH 1) with HCl (12 M). The compound was extracted into EtOAc and the combined extracts were evaporated under reduced pressure. The crude residue was purified by flash chromatography (hexane/EtOAc 4/1 + 1% HOAc) to isolate pure **13** (97.0 mg, quantitative isolated yield) as white solid (upon co-evaporation with toluene, subsequent precipitation by adding hexane drops to a DCM solution, and finally drying under high vacuum after evaporation under reduced pressure). R_f_ (hexane/EtOAc 4/1 + 5 drops of HOAc, PMA) 0.26. ^1^H-NMR (300 MHz, CDCl_3_) *δ* 3.98 (1H, br s, 12β-H), 3.33 (1H, m, 3β-H), 2.39–2.33 (1H, br m), 2.32–2.20 (1H, br m), 0.98 (3H, d, *J* = 6.1, 21-CH_3_), 0.92 (3H, s, 19-CH_3_), 0.67 (3H, s, 18-CH_3_). ^13^C-NMR (APT, 125 MHz, CDCl_3_) *δ* 12.8 (18-CH_3_), 17.3 (21-CH_3_), 23.3 (19-CH_3_), 23.6 (CH_2_), 26.1 (CH_2_), 26.7 (CH_2_), 27.1 (CH_2_), 27.4 (CH_2_), 28.7 (CH_2_), 29.7 (CH_2_), 30.7 (CH_2_), 32.5 (CH_2_), 33.7 (CH), 34.2 (C), 35.1 (CH), 35.4 (CH_2_), 36.0 (CH), 42.4 (CH), 46.5 (C), 47.4 (CH), 48.2 (CH), 61.3 (3-CH), 73.1 (12-CH), 178.5 (COOH). ESI^−^-MS (250 °C, MeOH/H_2_O 4/1) calcd. for C_24_H_39_N_3_O_3_ 417.3 Da, found *m/z* (% rel. int.) 416.5 (100) [M − H]^−^, 833.4 (19) [2M − H]^−^, 856.6 (10) [2M − 2H + Na]^−^. The spectra are available in the Supporting Information: Figures S7–S9.

*O-Acetylation of compound* **13** → *3α-Azido,12α-acetoxy-5β-cholan-24-oic acid* (**10**): In a round bottomed flask (1 mL), compound **13** (39.4 mg, 0.0944 mmol) was readily dissolved and overnight stirred at room temperature (argon atmosphere) in a stock solution (290 µL added) containing Ac_2_O (730 µL), DMAP (2.4 mg) and pyridine (2.9 mL). Cooled in an ice bath, the reaction mixture was carefully acidified (pH 1) with HCl (1.2 M) and the crude compound (white suspension) thoroughly extracted (100 mL funnel) into DCM. The combined extracts were evaporated under reduced pressure and the resulting colourless oil purified by flash chromatography (hexane/EtOAc 9/1 + 1% HOAc). Upon co-evaporation with toluene and drying under high vacuum, pure **10** (32.5 mg, 75% isolated yield) was obtained as a colorless oil. R_f_ (hexane/EtOAc 4/1 + 5 drops of HOAc, PMA) 0.11. ^1^H-NMR (500 MHz, CDCl_3_) *δ* 5.01 (1H, s, 12β-H), 3.19 (1H, m, 3β-H), 2.36–2.28 (1H, br m), 2.21–2.13 (1H, br m), 0.87 (3H, s, 19-CH_3_), 0.79 (3H, d, *J* = 6.1, 21-CH_3_), 0.67 (3H, s, 18-CH_3_). ^13^C-NMR (APT, 125 MHz, CDCl_3_) *δ* 12.5 (18-CH_3_), 17.5 (21-CH_3_), 21.3 (acetate CH_3_), 23.1 (19-CH_3_), 23.4 (CH_2_), 25.5 (CH_2_), 25.9 (CH_2_), 26.5 (CH_2_), 26.9 (CH_2_), 27.3 (CH_2_), 30.6 (CH_2_), 31.0 (C), 32.3 (CH_2_), 34.1 (CH_2_), 34.4 (CH), 34.7 (CH), 35.2 (CH_2_), 35.6 (CH), 42.2 (CH), 45.0 (C), 47.6 (CH), 49.4 (CH), 61.0 (CH), 75.9 (CH), 170.6 (acetate COOR), 179.6 (COOH). ESI^−^-MS (150 °C, MeOH/H_2_O 4/1 + 0.1% formic acid) calcd. for C_26_H_41_N_3_O_4_ 459.3 Da, found *m/z* (% rel. int.) 415.4 (8) [M − N_2_ − OH]^−^, 458.4 (87) [M − H]^−^, 504.2 (70) [M + formate]^−^, 874.4 (26) [2M − N2 − OH]^−^, 918.3 (100) [2M − H]^−^, 1332.6 (10) [3M − N2 − OH]^−^, 1376.8 (50) [3M − H]^−^. The spectra are available in the Supporting Information: Figures S10–S12.

*Coupling of building block ***10** *to H_2_N-photolinker-Tentagel* → *Ligation scaffold* **14**: The H_2_N-photolinker-Tentagel resin (155.1 mg, 0.19 mmol/g) was suspended in dry DMF (1350 µL), followed by addition of steroid scaffold **10** (16.2 mg, 0.035 mmol, 1.2 eq.), dry DIPEA (12.3 µL, 2.4 eq.) and PyBOP (18.4 mg, 1.2 eq.). Shielded from light by foil wrapping, the argon flushed vessel was gently agitated at room temperature overnight. Excess reagents and solvent were removed by filtration under reduced pressure, the content washed with DMF, MeOH and DCM, and the resulting resin **14** dried under high vacuum. This procedure was repeated once. An analytical sample was photolytically cleaved for evaluation (365 nm in ACN for 3 h): ESI^+^-MS (250 °C, MeOH/H_2_O 4/1 + 5 mM NH_4_OAc) calcd. for C_26_H_42_N_4_O_3_ 458.3 Da, found *m/z *(% rel. int.) 478.5 (100) [M + NH_4_]^+^, 371.4 (91) [M − N_2_ − OAc]^+^. The spectrum is available in the Supporting Information: Figure S13.

### 3.3. Synthesis of Scaffold ***15***

*Coupling of FmocHN-GABA-OH to 2-chlorotrityl resin, Fmoc deprotection and coupling of FmocHN-(L)-Pra-OH → Intermediate* **17**: To a suspension of 2-chlorotrityl resin (0.115 g, 1.55 mmol/g, 0.18 mmol) in dry DCM (1.5 mL), FmocHN-GABA-OH (0.12 g, 0.36 mmol) and DIPEA (0.37 mL, 2.14 mmol) were added. The reaction mixture was shaken at room temperature for 3 h, after which reagents and solvent were removed by filtration under reduced pressure. Washed with NMP, MeOH, DCM and Et_2_O, the resin was dried under high vacuum. By Fmoc-UV measurements [55], the loading was determined to be 0.89 mmol/g, which gave a coupling yield of 87%. This resin (0.12 g, 0.89 mmol/g) was Fmoc deprotected by double treatment with a solution of 20% piperidine/DMF for 20 min (intermediate filtration). Washed with DMF and NMP, FmocHN-(L)-Pra-OH (0.16 g, 0.48 mmol, 0.5 M in NMP), PyBOP (0.25 g, 0.48 mmol, 0.5 M in NMP) and DIPEA (0.17 mL, 0.97 mmol, 2 M in NMP) were added to a suspension in NMP, and the reaction mixture was shaken at room temperature for 2 h. Reagents and solvent were removed by filtration under reduced pressure. Washed with NMP, MeOH, DCM and Et_2_O, the resin was dried under high vacuum. An analytical sample was acidolytically cleaved for evaluation (AcOH/TFE/DCM 1/1/3 at RT for 2 h, followed by co-evaporation of the filtrate with toluene under reduced pressure): LC-MS (C18 100 Å, 0 to 100% B in 15 min, 214 nm) *t_ret_* 12.6 min → (pos. mode, 250 °C) calcd. for C_24_H_24_N_2_O_5_ 420.2 Da, found *m/z* (% rel. int.) 421.1 (100) [M + H]^+^. A figure including the chromatogram and spectrum is available in the Supporting Information: Figure S14.

*Fmoc deprotection, coupling of building block* **3**, *Alloc deprotection and coupling of azidoacetic acid → Ligation scaffold ***15**: Resin **17** (0.11 g, 0.89 mmol/g) was subjected to Fmoc deprotection by treatment with a 20% piperidine/DMF solution (1 mL) for 1, 5 and 8 min (intermediate filtration). Washed with NMP, MeOH, DCM and Et_2_O and dried under high vacuum, this resin was resuspended in NMP, followed by addition of steroid scaffold **3** (0.18 g, 0.29 mmol, 0.5 M in NMP), PyBOP (0.15 g, 0.29 mmol, 0.5 M in NMP) and DIPEA (0.10 mL, 0.581 mmol, 2 M in NMP). The reaction mixture was shaken at room temperature for 2 h. Reagents and solvent were removed by filtration under reduced pressure. The resin was washed with NMP, MeOH, DCM and Et_2_O, and dried under high vacuum. Resuspended in dry DCM (300 µL), Alloc deprotection was performed by treatment with PhSiH_3_ (0.36 mL, 2.9 mmol) and Pd(Ph_3_P)_4_ (11.2 mg, 0.009 mmol) at room temperature for 1 h. Reagents and solvent were removed by filtration under reduced pressure, the resin was washed with dry DCM, and the deprotection procedure repeated twice. Finally, the resin was further washed with NMP, MeOH, DCM and Et_2_O, and dried under high vacuum. Resuspended in NMP, azidoacetic acid (0.05 g, 0.49 mmol, 0.5 M in NMP), PyBOP (0.25 g, 0.49 mmol, 0.5 M in NMP) and DIPEA (0.17 mL, 0.97 mmol, 2 M in NMP) were added to the resulting resin (0.11 g, 0,89 mmol). The reaction mixture was shaken at room temperature for 3 h, reagents and solvent were removed by filtration under reduced pressure, and the resin was washed with NMP, MeOH, DCM and Et_2_O. The same procedure was repeated once, and the resulting resin **15** dried under high vacuum. An analytical sample was acidolytically cleaved for evaluation (AcOH/TFE/DCM 1/1/3 at RT for 2 h, followed by co-evaporation of the filtrate with toluene under reduced pressure): LC-MS (C18 100 Å, 0 to 100% B in 15 min, 214 nm) *t_ret_* 13.6 min → (pos. mode, 250 °C) calcd. for C_42_H_65_N_7_O_9_ 811.5 Da, found *m/z* (% rel. int.) 712.3 (100) [M − Boc]^+^, 812.4 (15) [M + H]^+^. A figure including the chromatogram and spectrum is available in the Supporting Information: Figure S15.

### 3.4. Synthesis of Scaffold ***22***

*Coupling of BocHN-(L)-Cys(Trt)-OH to* **19** → *Intermediate ***20** [53]: Resin **19** (50 mg, 0.23 mmol/g, 0.0115 mmol) was suspended in a DCM/DMF_dry_ (1/1 v/v, 1 mL) mixture. During pre-swelling (~45 min) of the resin, BocHN-(L)-Cys(Trt)-OH (21.3 mg, 4 eq.) was weighed in a round-bottomed, argon flushed flask (10 mL), and dissolved in a DCM/DMF_dry_ mixture (1/1 v/v, 400 μL). HOBt (6.3 mg, 4 eq.), DCM/DMF_dry_ (1/1 v/v, 300 μL) and DIC (7.0 μL, 4 eq.) were consecutively added. Rapid dissolution was observed, and the resulting mixture was manually swirled for ~5 min, until initial formation of solid particles was observed. Meanwhile, the resin was filtered under reduced pressure. The pre-activated mixture was transferred to the resin, aided by additional DCM/DMF_dry_ (1/1 v/v, 700 μL) solvent. The resulting coupling mixture was flushed with argon and shaken overnight at room temperature, shielded from light. Excess reagents and solvents were removed under reduced pressure and the resin was washed with DMF, MeOH and DCM, after which the procedure was repeated. The obtained resin **20** was dried under high vacuum. An analytical sample was photolytically cleaved for evaluation (365 nm in ACN for 3 h): RP-HPLC (C_18_ 100 Å, 75 to 100% B in 15 min, 214 and 254 nm) *t_ret_* 17.3 min. ESI^+^-MS (250 °C, MeOH/H_2_O 4/1) calcd. for C_55_H_74_N_4_O_6_S 918.5 Da, found *m/z* (% rel. int.) 918.9 (28) [M + H]^+^, 941.4 (100) [M + Na]^+^, 957.4 (11) [M + K]^+^, 841.4 (6) [M − Boc + Na]^+^, 1837.9 (19) [2M + H]^+^, 1859.2 (22) [2M + Na]^+^, 243.2 (67) [Trt + H]^+^. The chromatogram and spectrum are available in the Supporting Information: Figures S16–S17.

*Tuned C3-NHAlloc deprotection of* **20** → *Intermediate ***21**: Resin **20** (~0.0115 mmol) was suspended in DCM (1.5 mL), the reactor flushed with argon, and morpholine (180 μL, 180 eq.), Bu_3_SnH (60 μL, 20 eq.) and Pd(PPh_3_)_4_ (20 mg, 1.5 eq.) were sequentially added, followed by DCM (1.5 mL). Flushed with argon and shielded from light, the orange deprotection mixture was shaken overnight at room temperature. Excess reagents and solvent were removed by filtration under reduced pressure, and the resin beads washed with DMF, MeOH and DCM. The obtained resin **21** was dried under high vacuum. An analytical sample was photolytically cleaved for evaluation (365 nm in ACN for 3 h): RP-HPLC (C_18_ 100 Å, 0 to 100% B in 15 min, 214 and 254 nm) *t_ret_* 17.5 min. ESI^+^-MS (250 °C, MeOH/H_2_O 4/1) calcd. for C_51_H_70_N_4_O_4_S 834.5 Da, found *m/z* (% rel. int.) 835.2 (100) [M + H]^+^, 857.4 (96) [M + Na]^+^, 873.3 (21) [M + K]^+^, 1670.2 (17) [2M + H]^+^, 1692.1 (38) [2M + Na]^+^, 243.2 (54) [Trt + H]^+^. The chromatogram and spectrum are available in the Supporting Information: Figures S18–S19.

*Coupling of FmocHN-(L)-Cys(Trt)-OH to* **21** → *Ligation scaffold* **22** [53]: By adopting the same procedure (*vide supra*) for manual introduction of BocHN-(L)-Cys(Trt)-OH residue at the initial C12-position, the Fmoc-protected counterpart was attached to the C3-position of resin-bound steroid scaffold **21**, yielding final resin **22**. An analytical sample was photolytically cleaved for evaluation (365 nm in ACN for 3 h): RP-HPLC (C_18_ 100 Å, 75 to 100% B in 15 min, 214 and 254 nm) *t_ret_* 20.4 min. ESI^+^-MS (250 °C, MeOH/H_2_O 4/1) calcd. for C_88_H_99_N_5_O_7_S_2_ 1401.7 Da, found *m/z* (% rel. int.) 1424.5 (100) [M + Na]^+^, 1440.3 (10) [M + K]^+^, 1303.4 (5) [M − Boc + H]^+^, 1324.5 (7) [M − Boc + Na]^+^, 243.2 (62) [Trt + H]^+^. The chromatogram and spectrum are available in the Supporting Information: Figures S20–S21.

## 4. Conclusions

Bile acid-peptide conjugates and macrocycles are accessible through a variety of solid-phase procedures. To enable decoration of the bile acid scaffold via convergent ligation methods rather than long linear SPPS procedures, particular reactive handles for chemoselective conversion have been introduced in various ways. In first instance, we show that based on an intercepted route towards Davis’ template **3**, or our template **4**, providing a combined click/alkylation (acylation) bile acid for double convergent ligation, a first hurdle towards that aim has been taken. Furthermore, on either the C3 + C24 (external), the C3 + C12 or a combination of these positions, we here illustrate that a variety of handles can be introduced towards application of Staudinger, click and native chemical ligation for the convergent construction of multivalent heteromeric conjugates. Expanding the toolbox of cholic acid based building blocks, the here described templates allow for modularity and diversity and bring convergent ligation based solid-phase parallel library endeavors within reach.

## Supplementary Materials

Supplementary materials can be accessed on: http://www.mdpi.com/1420-3049/16/12/10168/s1.
