# Supplementary material for: Shortcut Access to Peptidosteroid Conjugates: Building Blocks for Solid-Phase Bile Acid Scaffold Decoration by Convergent Ligation
Source: Molecules. 2011 Dec 7;16(12):10168–86. doi: 10.3390/molecules161210168 (PMC6264362; doi:10.3390/molecules161210168)
Supplement: Supplementary file 1 [file molecules-16-10168-s001.pdf]

*1. Synthesis of Scaffold **14***

Methyl 3α,12α-dihydroxy-5β-cholan-24-oate (11)

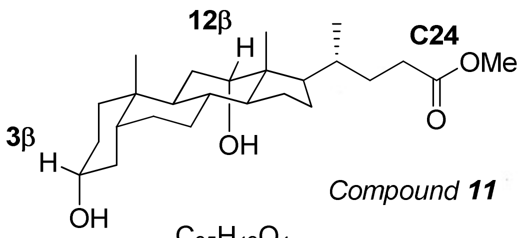

*Compound **11***

$C_{25}H_{42}O_4$
Exact Mass: 406.3
Molecular Weight: 406.6

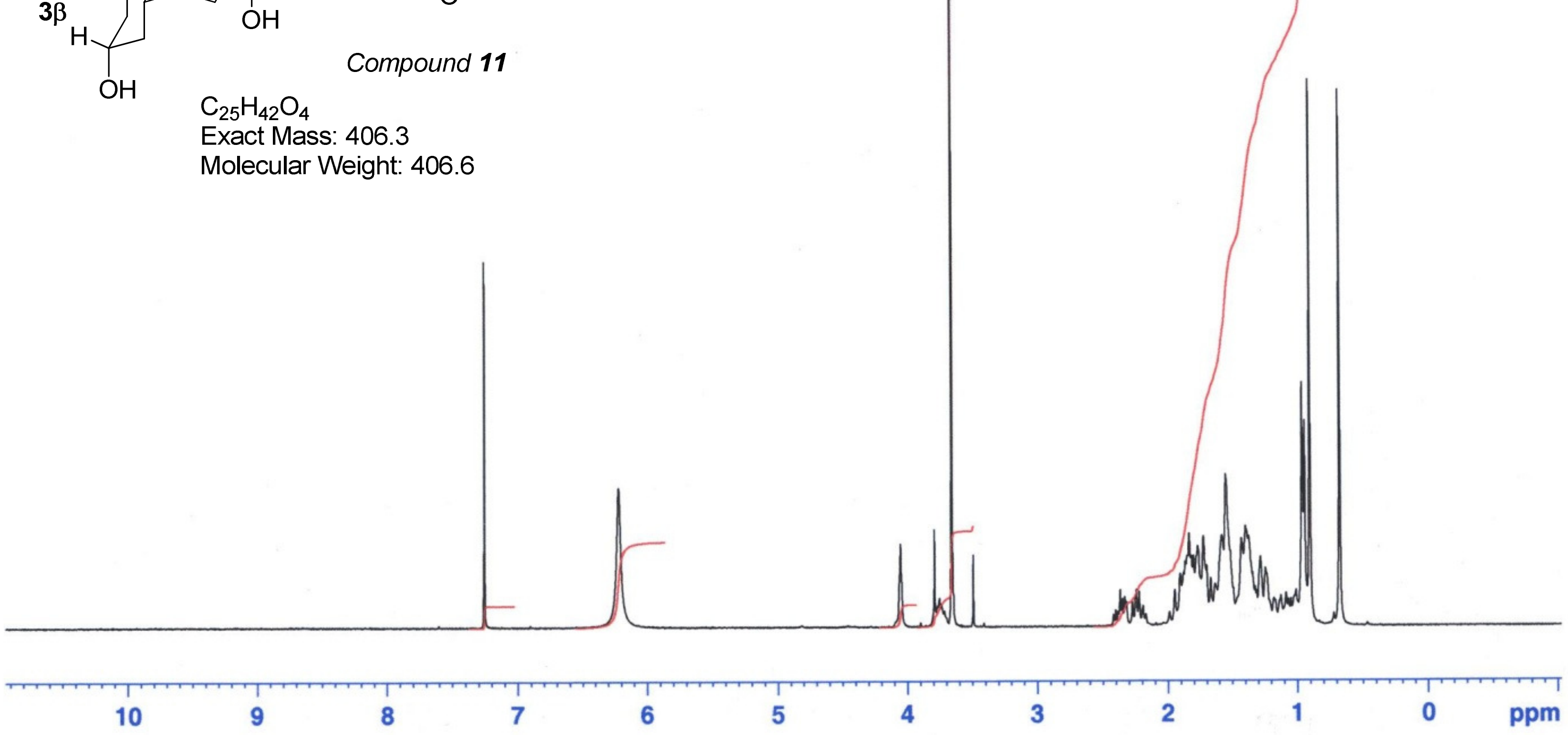

Figure S1. ^{1}H-NMR of compound **11**.

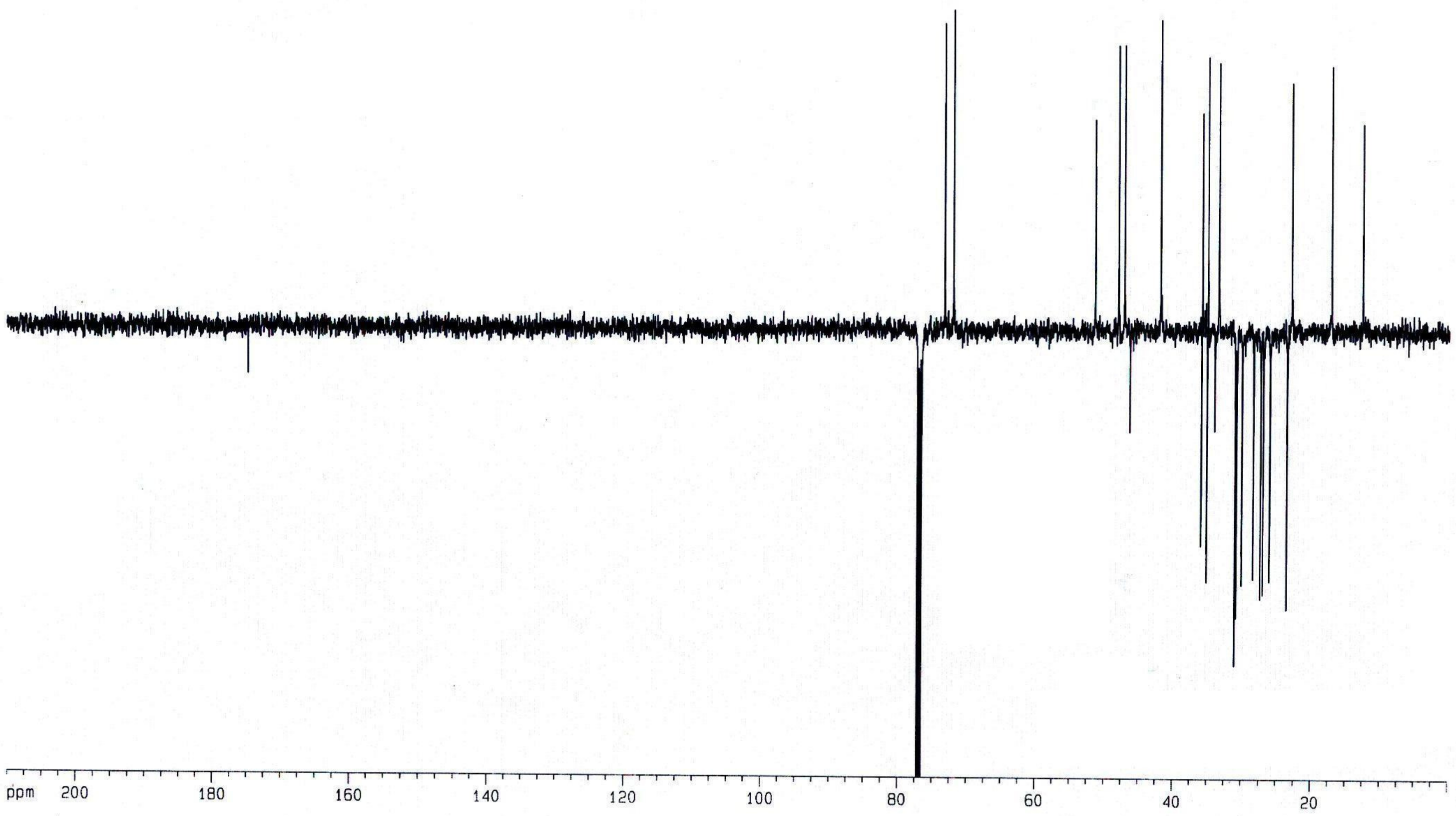

Figure S2. ^{13}C-NMR (APT) of compound **11**.

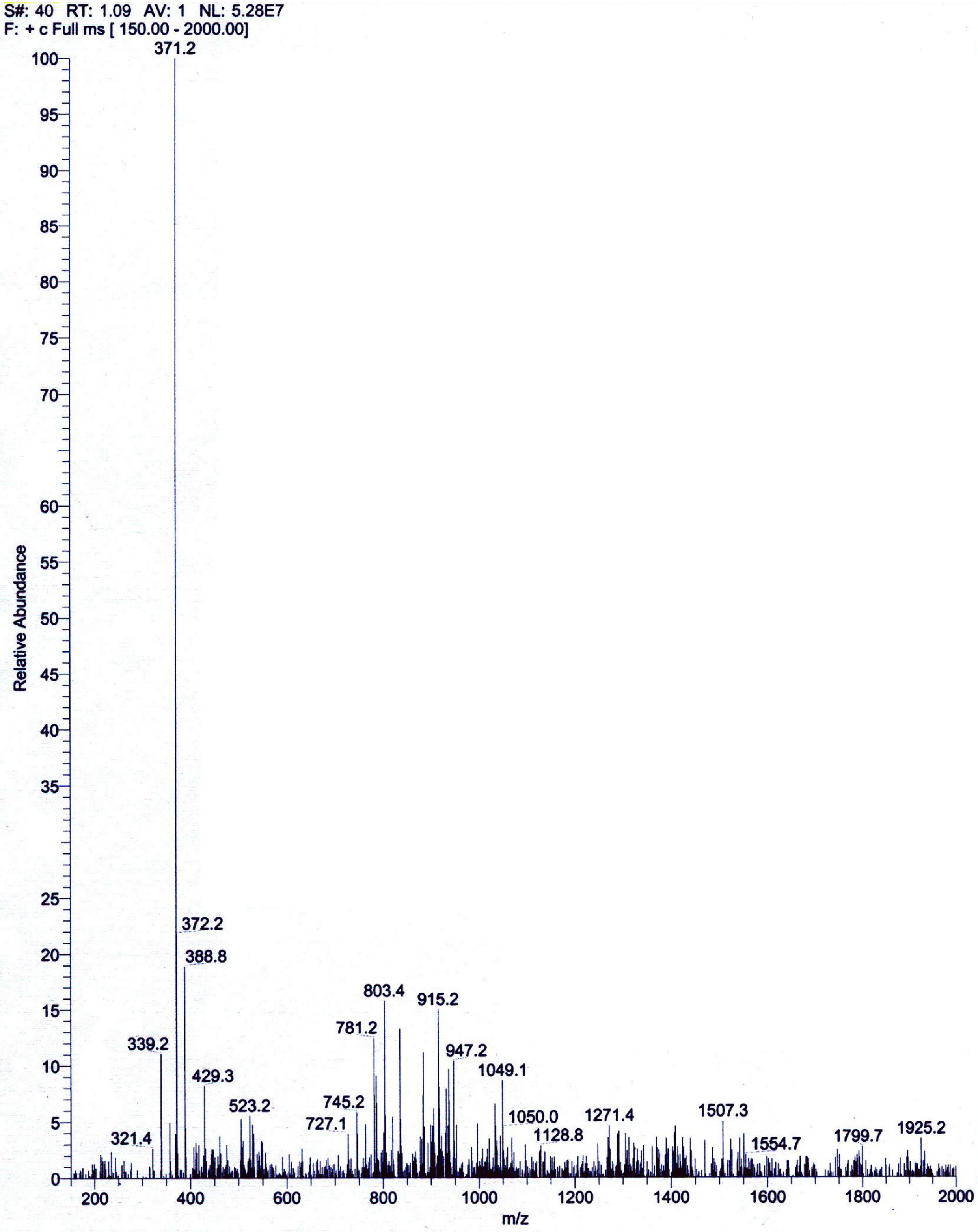

Figure S3. ESI-MS of compound **11**.

Methyl 3α-azido,12α-hydroxy-5β-cholan-24-oate (12)

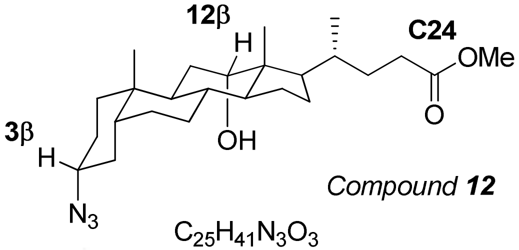

Compound ***12***

$C_{25}H_{41}N_3O_3$
Exact Mass: 431.3
Molecular Weight: 431.6

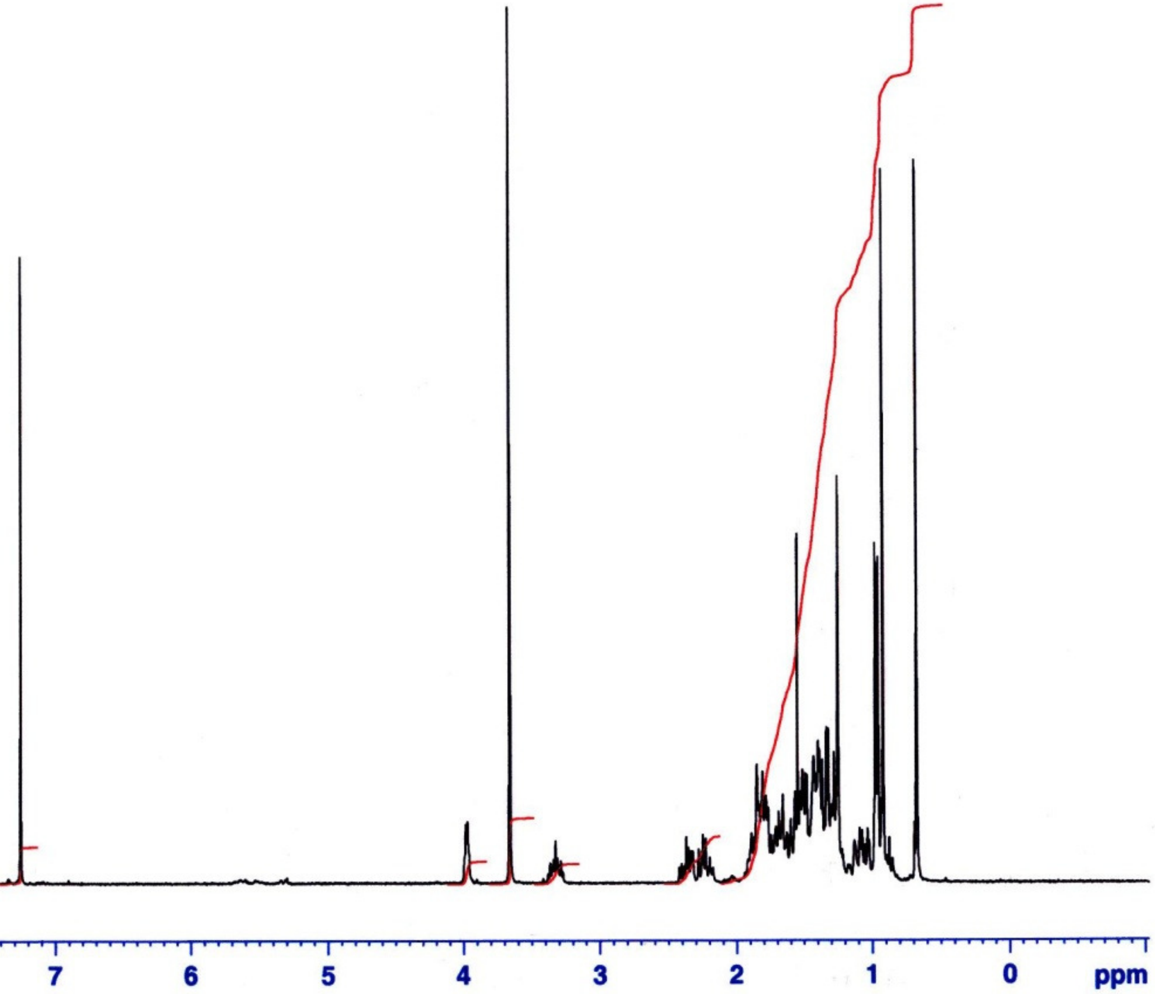

Figure S4. ^{1}H-NMR of compound **12**.

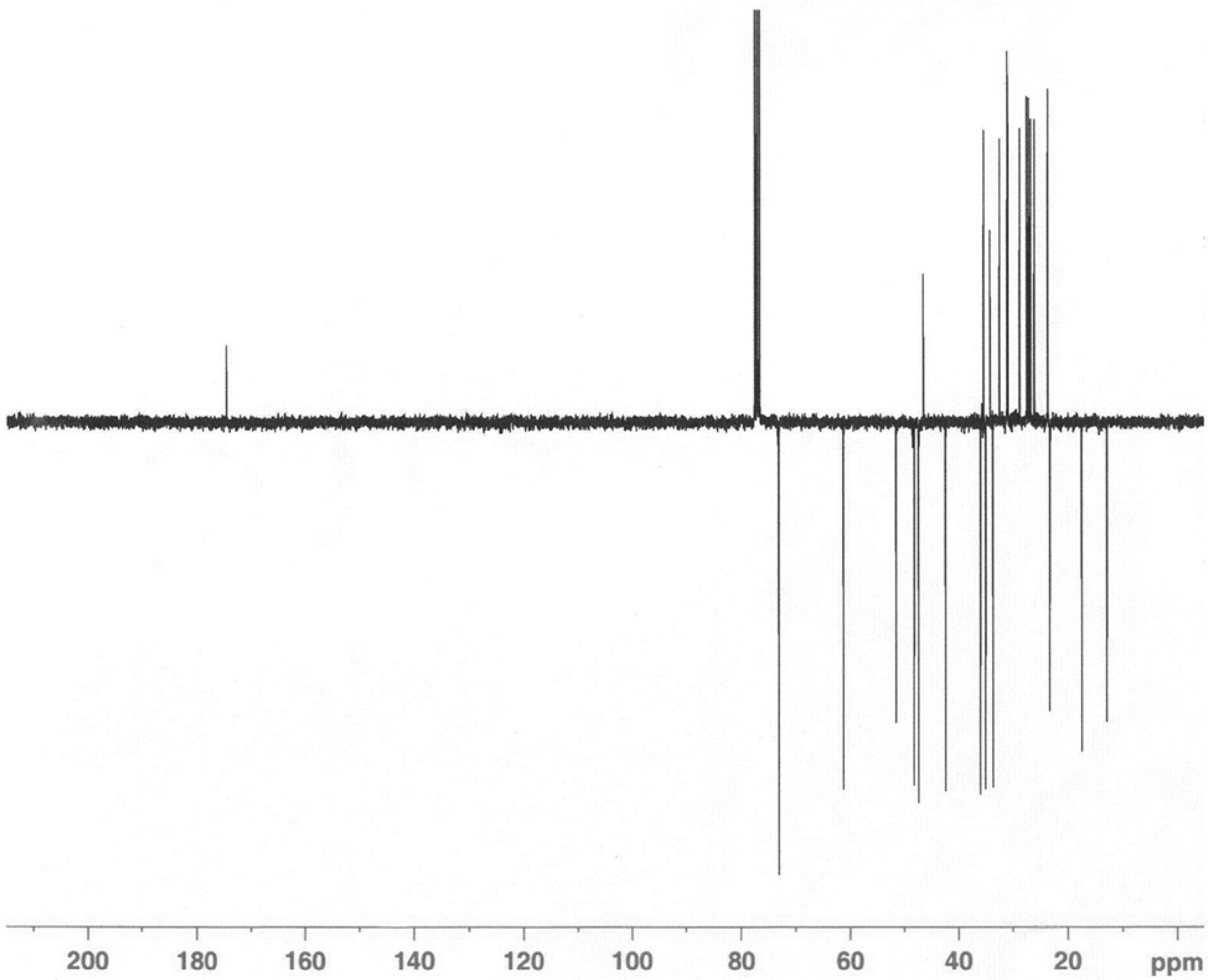

Figure S5. ^{13}C-NMR (APT) of compound **12**.

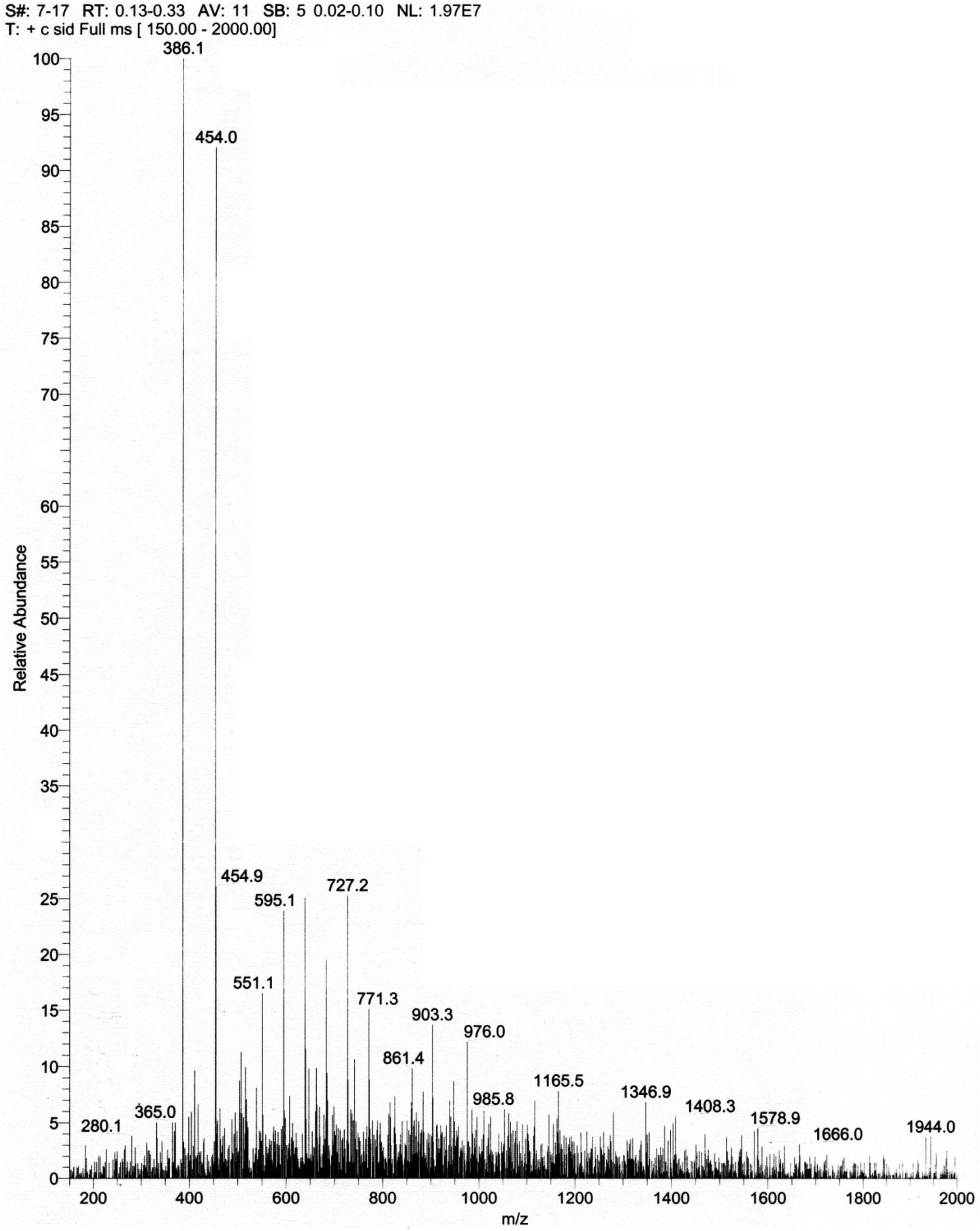

Figure S6. ESI-MS of compound **12**.

<u>3α-Azido,12α-hydroxy-5β-cholan-24-oic acid (13)</u>

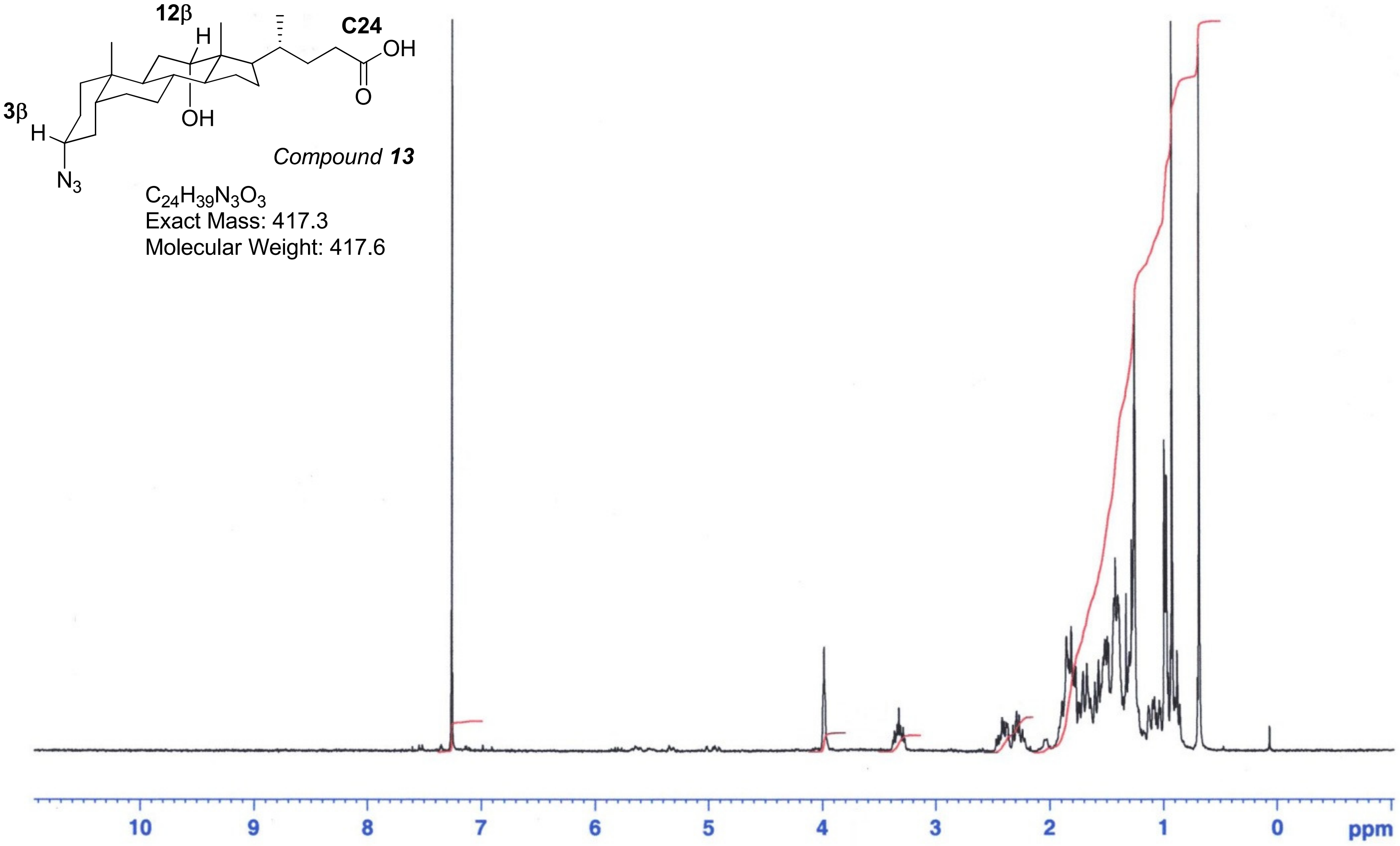

*Compound **13***

$C_{24}H_{39}N_3O_3$
Exact Mass: 417.3
Molecular Weight: 417.6

Figure S7. ^{1}H-NMR of compound **13**.

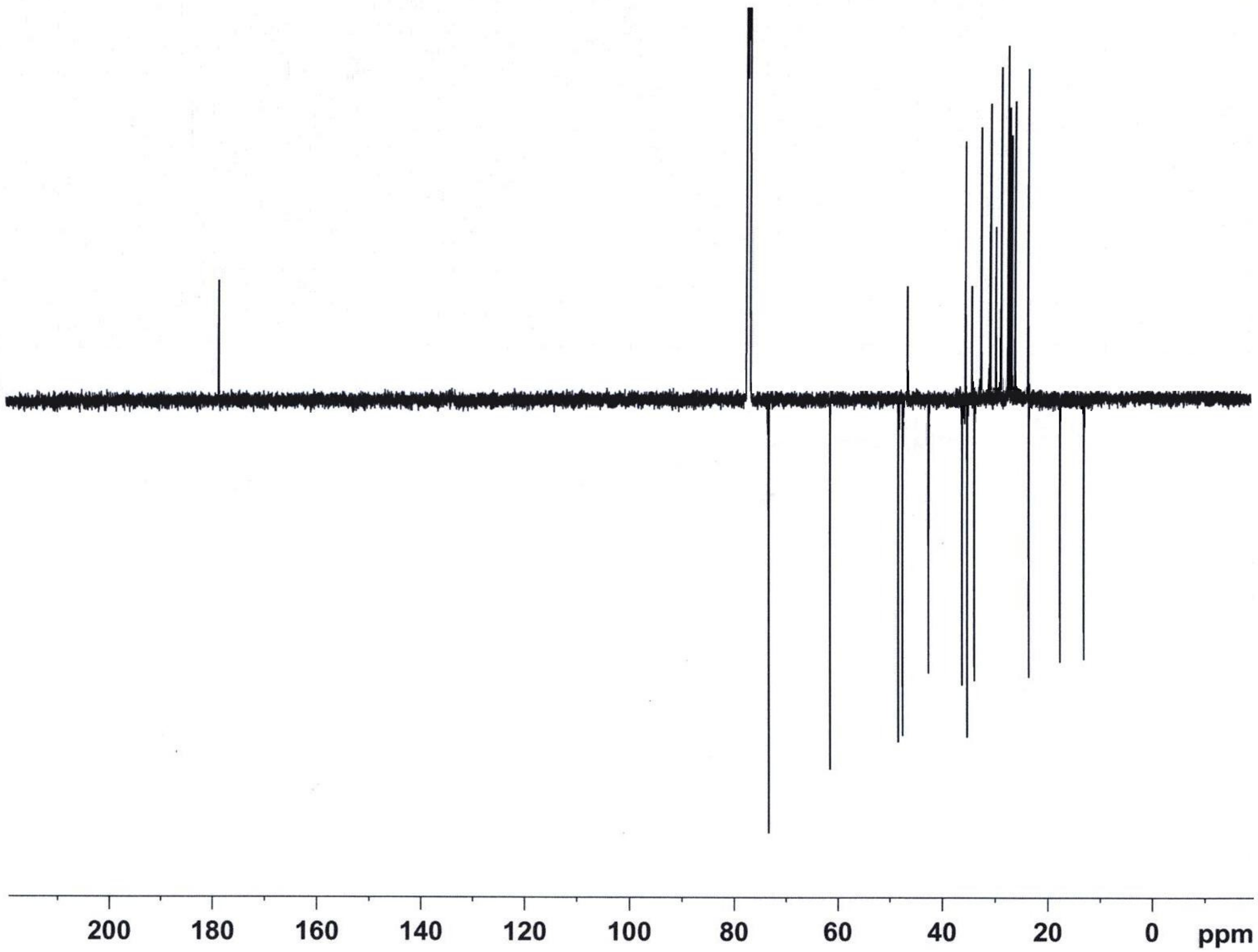

Figure S8. ^{13}C-NMR (APT) of compound **13**.

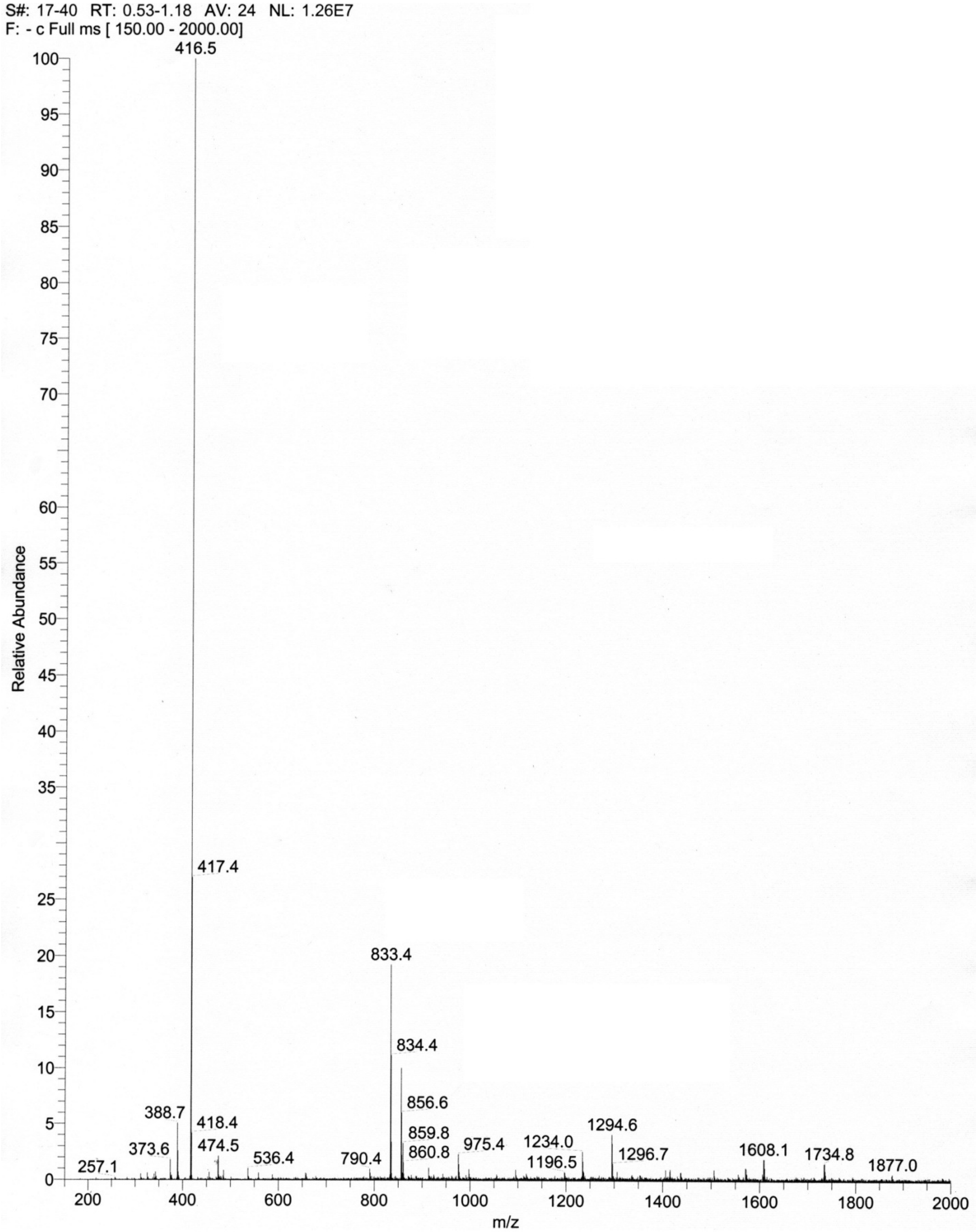

Figure S9. ESI-MS of compound **13**.

__3α-Azido,12α-acetoxy-5β-cholan-24-oic acid (10)__

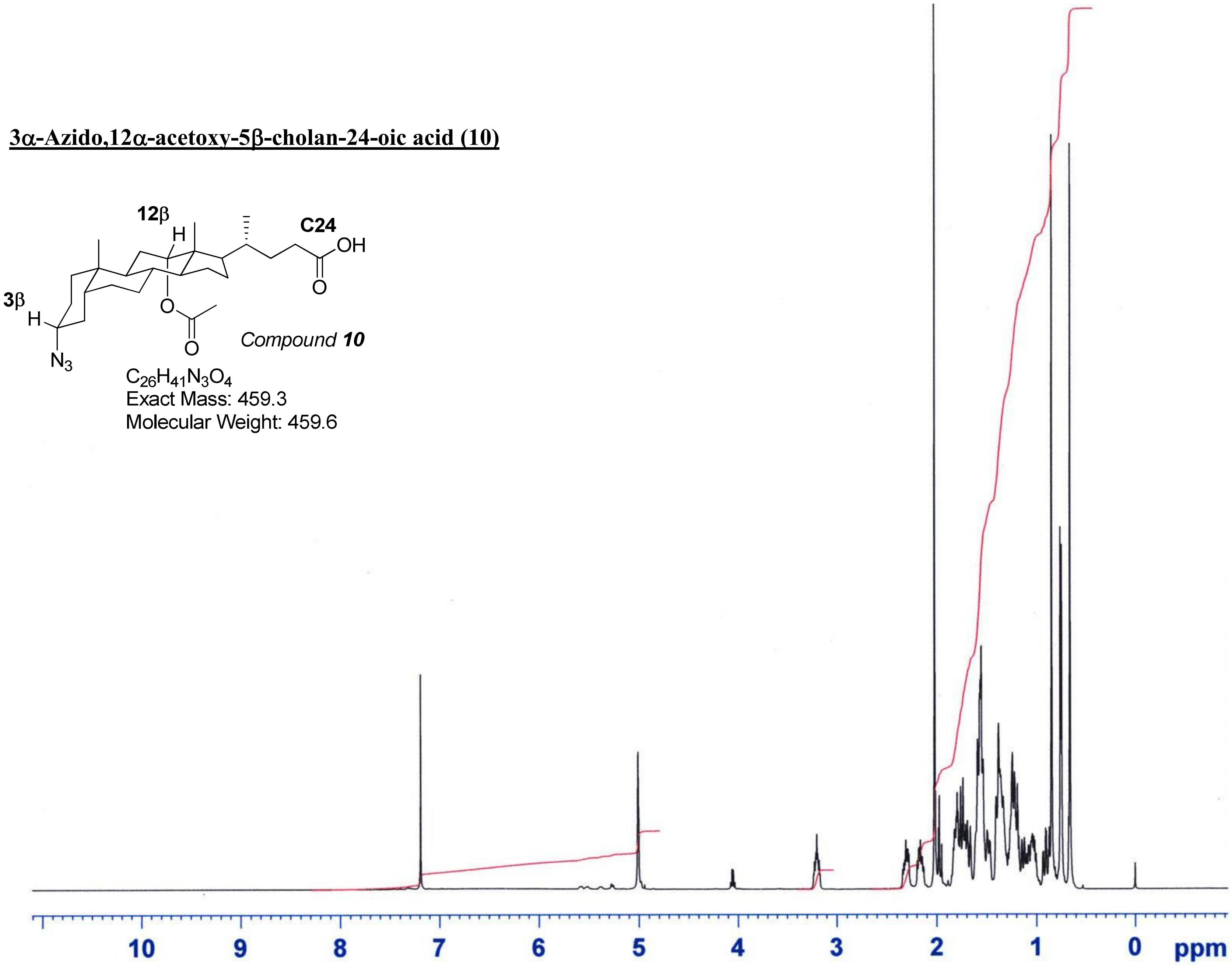

Compound ***10***

$C_{26}H_{41}N_3O_4$
Exact Mass: 459.3
Molecular Weight: 459.6

Figure S10. ^{1}H-NMR of compound **10**.

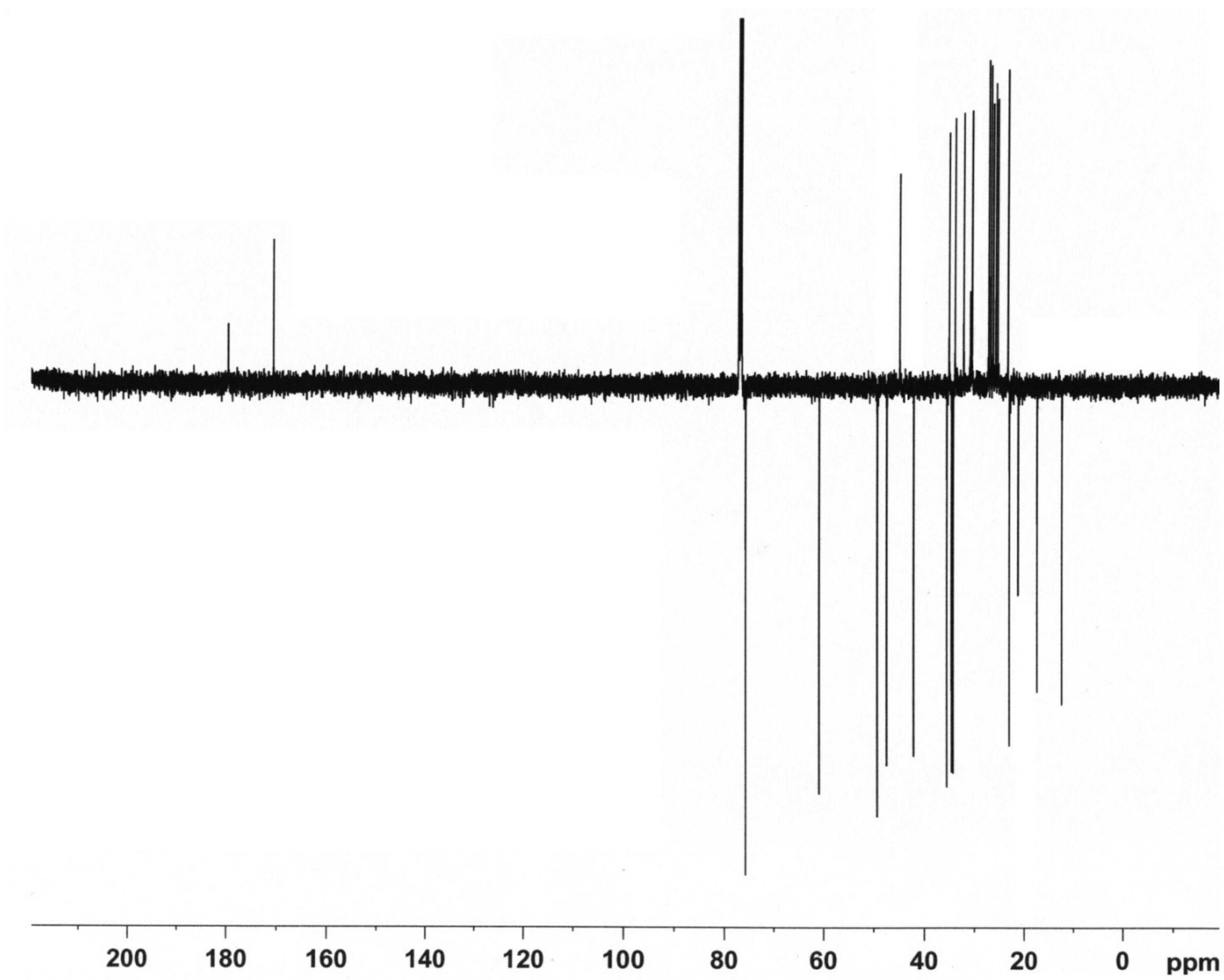

Figure S11. ^{13}C-NMR (APT) of compound **10**.

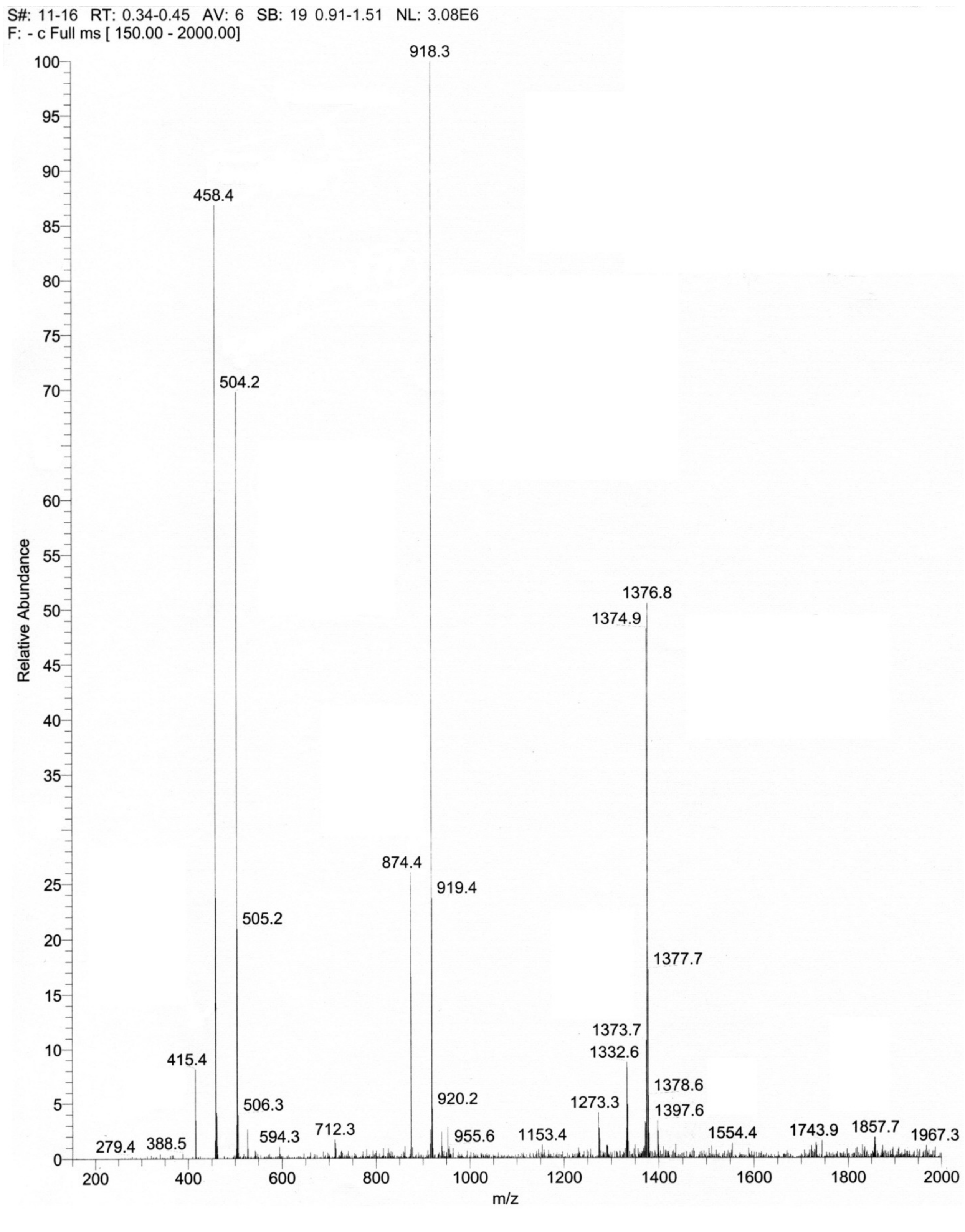

Figure S12. ESI-MS of compound **10**.

N_3-C3 decorated scaffold cleaved from 14

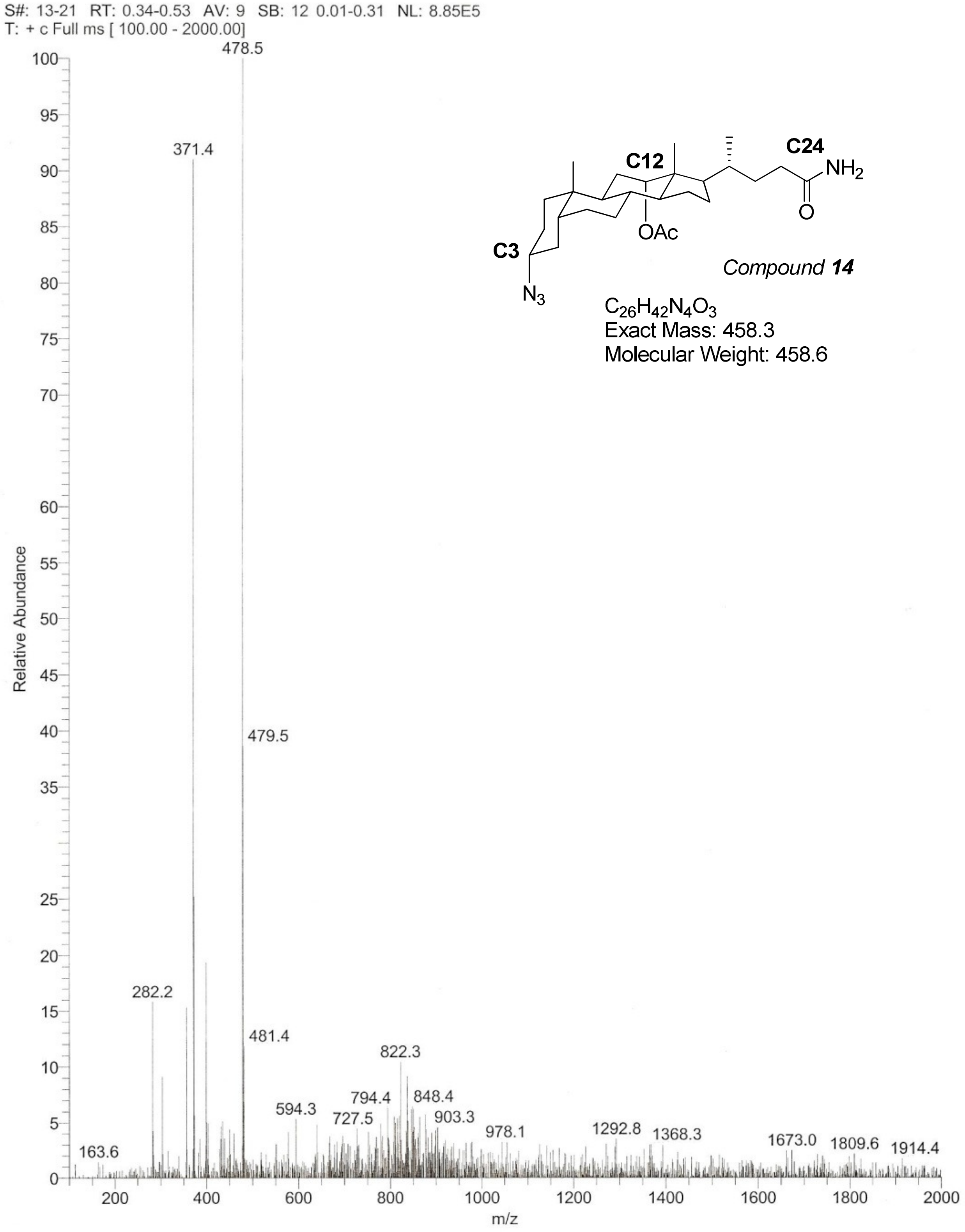

Figure S13. ESI-MS of compound **14**.

External FmocHN-(L)-Pra-GABA linker cleaved from 17

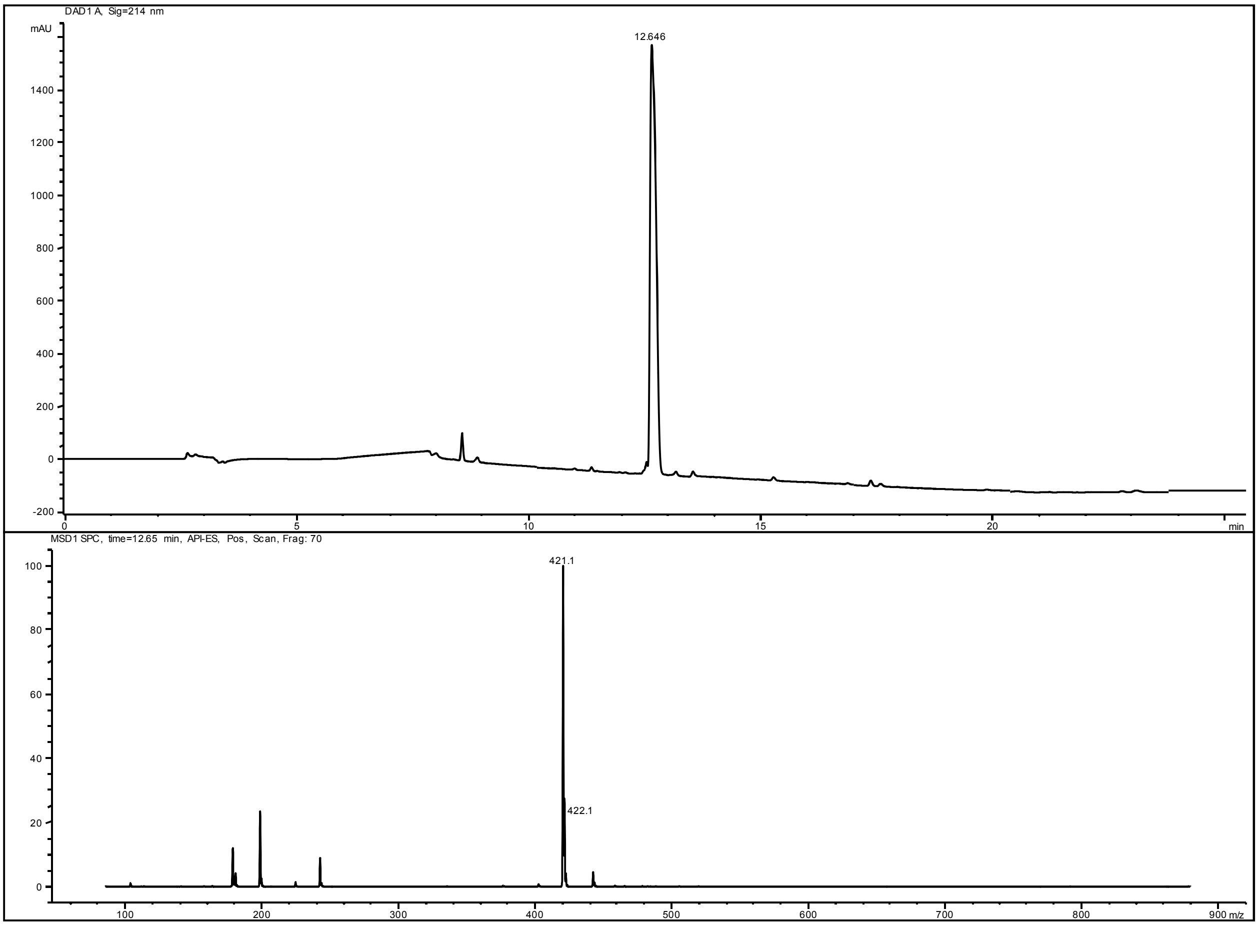

Figure S14. LC-MS of compound **17**.

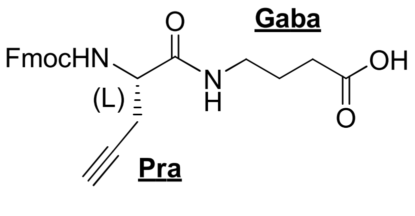

*Compound **17***

$C_{24}H_{24}N_2O_5$
Exact Mass: 420.2
Molecular Weight: 420.5

N_3-Gly-C3, C24-(L)-Pra-GABA decorated scaffold cleaved from 15

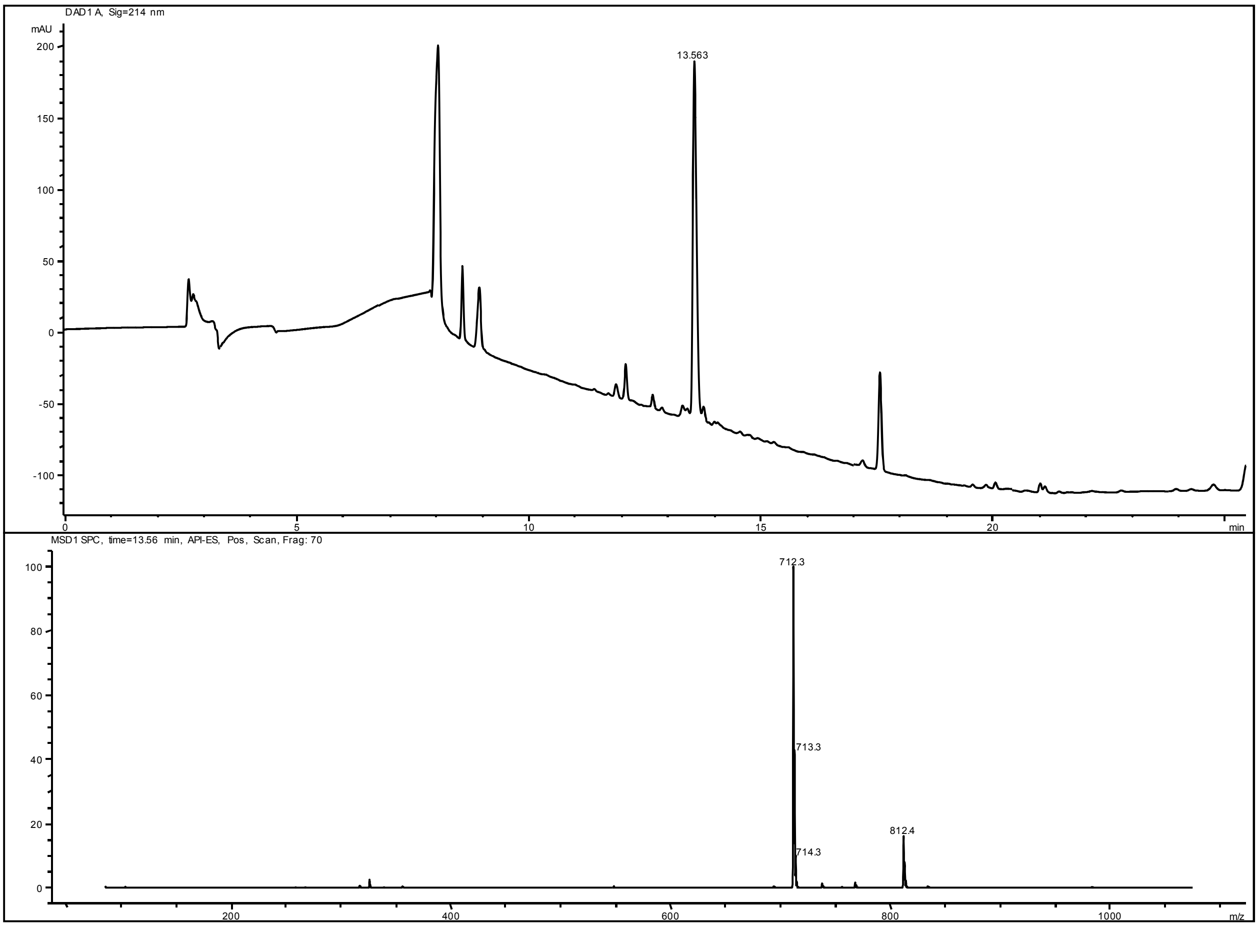

Figure S15. LC-MS of compound **15**.

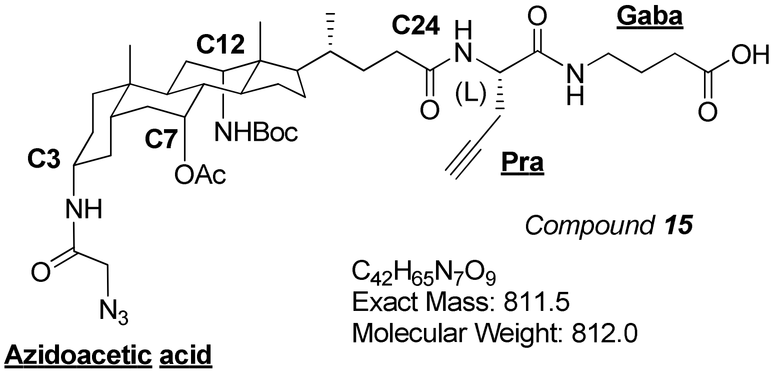

*Compound **15***

$C_{42}H_{65}N_7O_9$
Exact Mass: 811.5
Molecular Weight: 812.0

<u>AllocHN-C3, BocHN-(L)-Cys(Trt)-C12 decorated scaffold cleaved from 20</u>

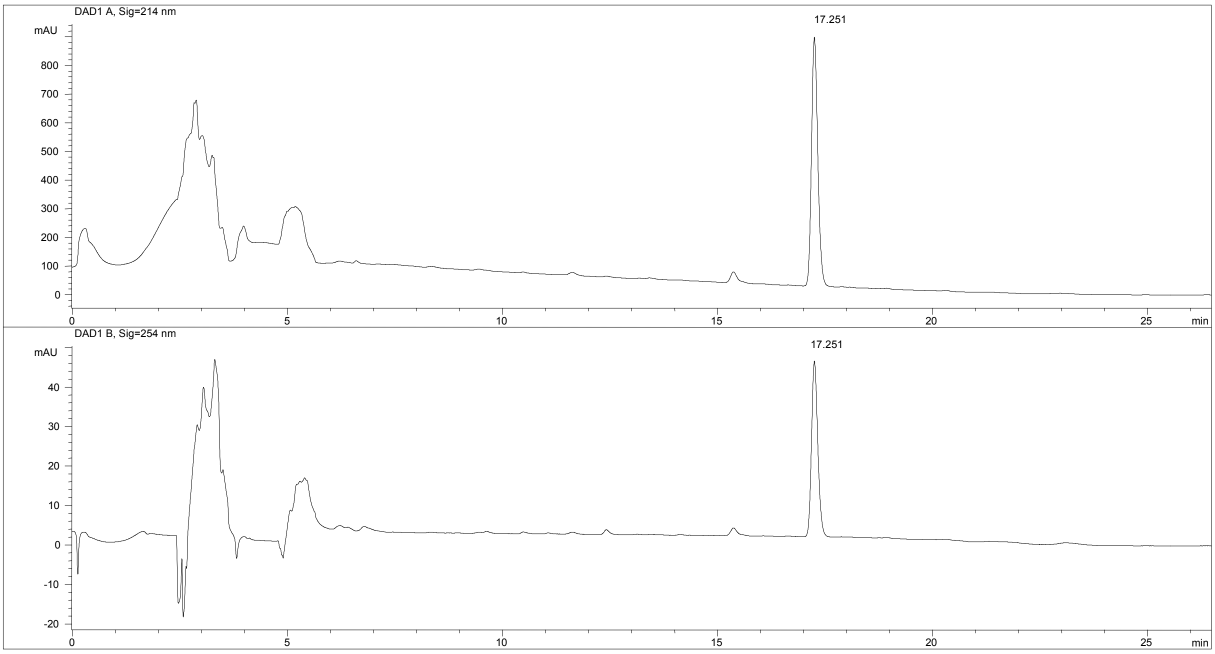

Figure S16. RP-HPLC of compound **20**.

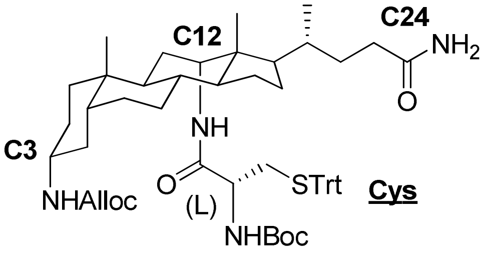

*Compound **20***

$C_{55}H_{74}N_4O_6S$
Exact Mass: 918.5
Molecular Weight: 919.3

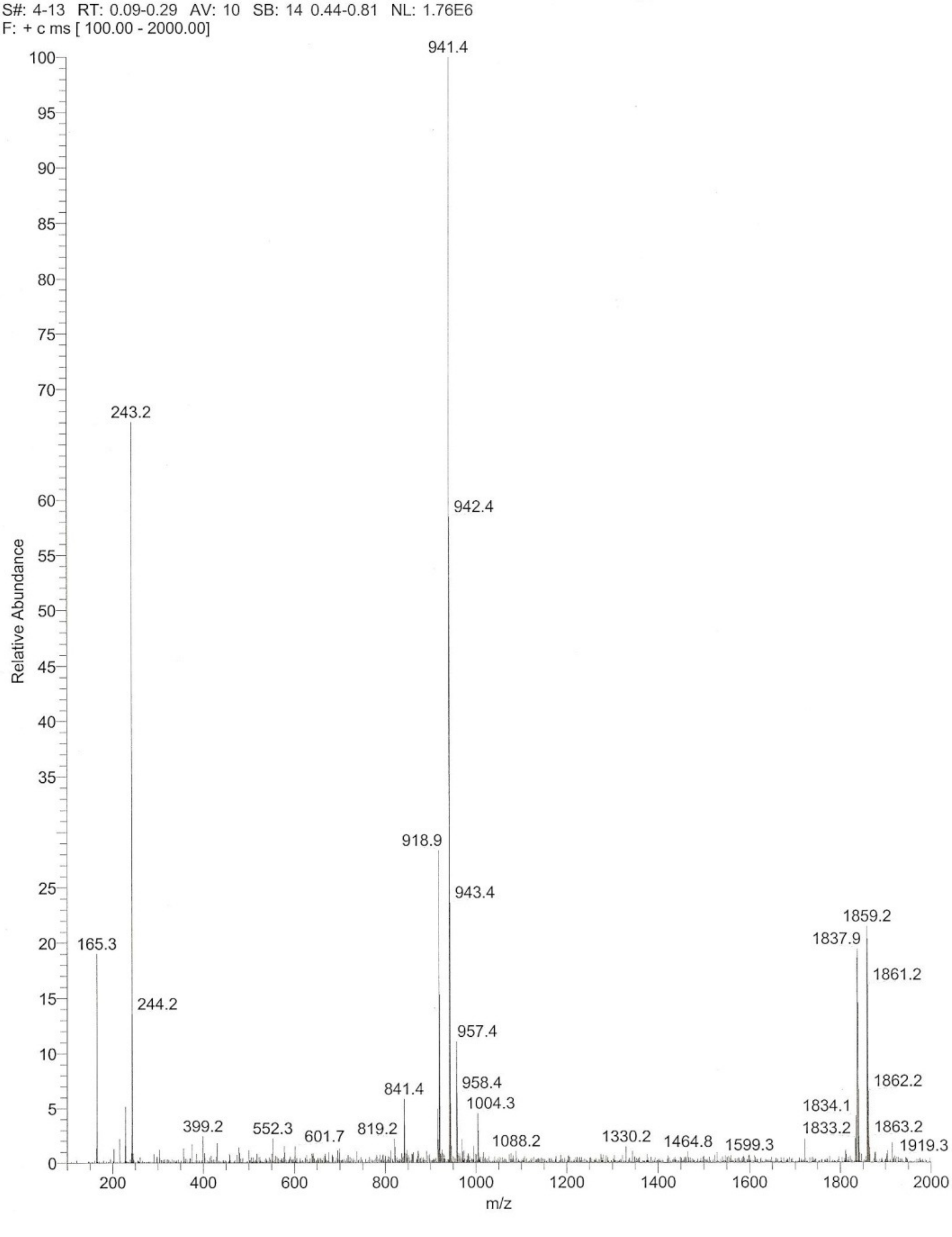

Figure S17. ESI-MS of compound **20**.

H$_2$N-C3, BocHN-(L)-Cys(Trt)-C12 decorated scaffold cleaved from 21

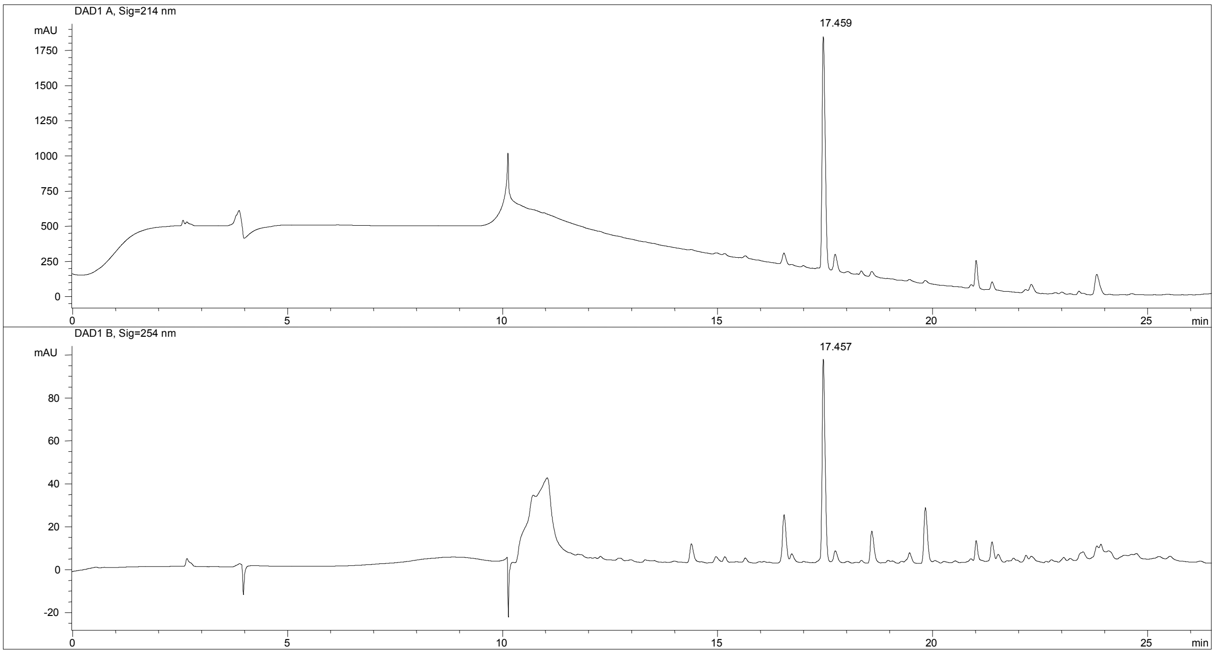

Figure S18. RP-HPLC of compound **21**.

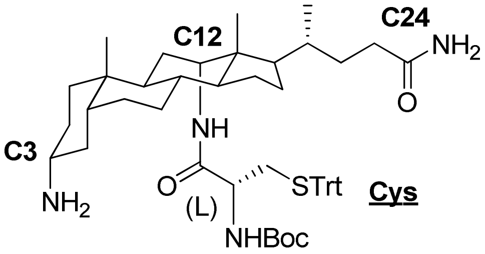

Compound ***21***

$C_{51}H_{70}N_4O_4S$
Exact Mass: 834.5
Molecular Weight: 835.2

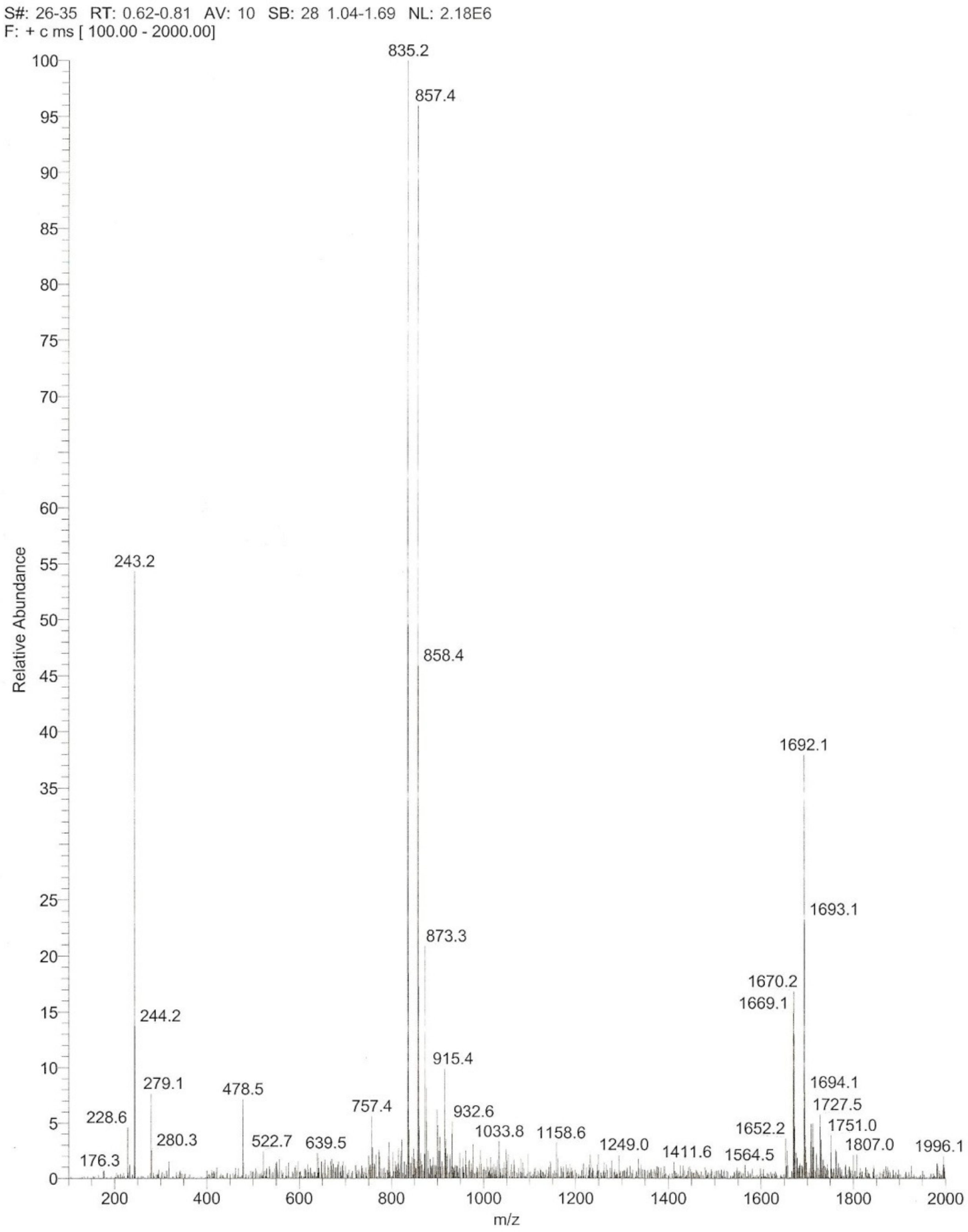

Figure S19. ESI-MS of compound **21**.

FmocHN-(L)-Cys(Trt)-C3, BocHN-(L)-Cys(Trt)-C12 decorated scaffold cleaved from 22

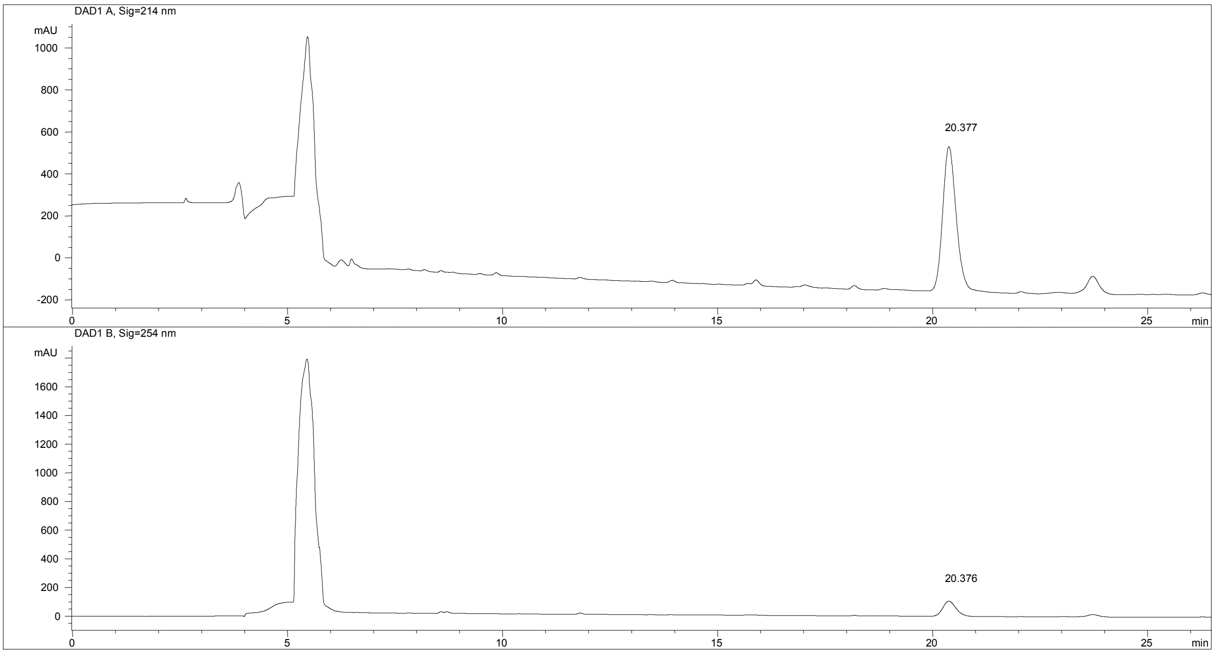

Figure S20. RP-HPLC of compound **22**.

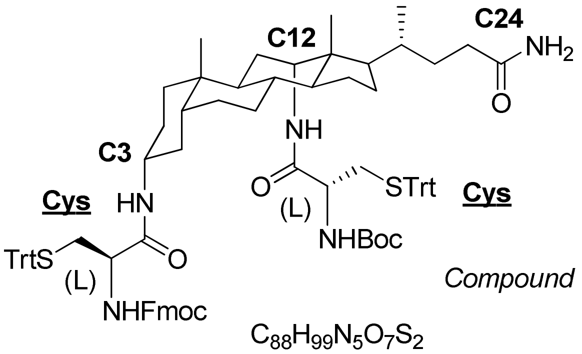

*Compound **22***

$C_{88}H_{99}N_5O_7S_2$
Exact Mass: 1401.7
Molecular Weight: 1402.9

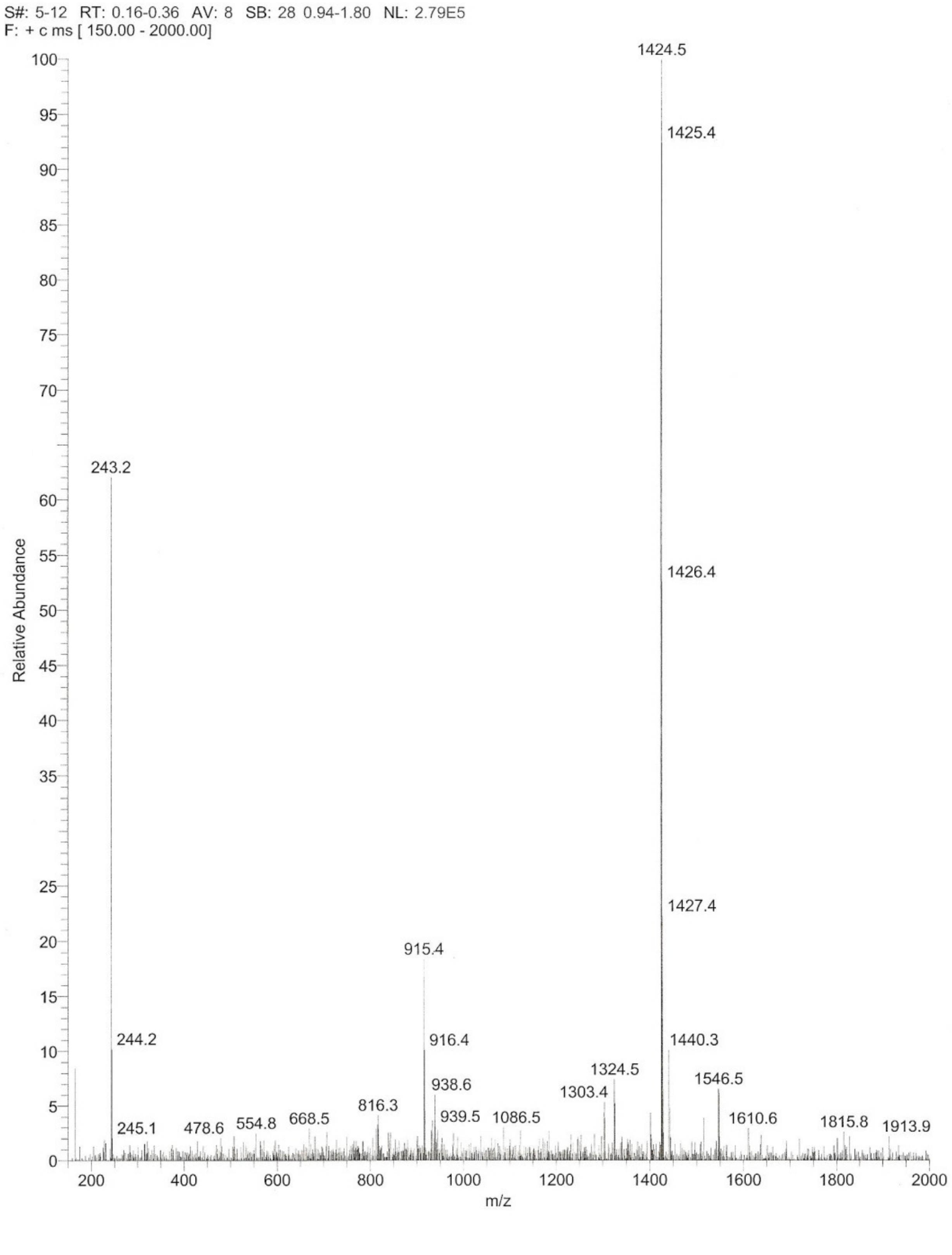

Figure S21. ESI-MS of compound **22**.
